# Discovering Biological Progression Underlying Microarray Samples

**DOI:** 10.1371/journal.pcbi.1001123

**Published:** 2011-04-14

**Authors:** Peng Qiu, Andrew J. Gentles, Sylvia K. Plevritis

**Affiliations:** 1 Department of Radiology, Stanford University, Stanford, California, United States of America; 2 Department of Bioinformatics and Computational Biology, The University of Texas MD Anderson Cancer Center, Houston, Texas, United States of America; University of Wisconsin-Madison, United States of America

## Abstract

In biological systems that undergo processes such as differentiation, a clear concept of progression exists. We present a novel computational approach, called Sample Progression Discovery (SPD), to discover patterns of biological progression underlying microarray gene expression data. SPD assumes that individual samples of a microarray dataset are related by an unknown biological process (i.e., differentiation, development, cell cycle, disease progression), and that each sample represents one unknown point along the progression of that process. SPD aims to organize the samples in a manner that reveals the underlying progression and to simultaneously identify subsets of genes that are responsible for that progression. We demonstrate the performance of SPD on a variety of microarray datasets that were generated by sampling a biological process at different points along its progression, without providing SPD any information of the underlying process. When applied to a cell cycle time series microarray dataset, SPD was not provided any prior knowledge of samples' time order or of which genes are cell-cycle regulated, yet SPD recovered the correct time order and identified many genes that have been associated with the cell cycle. When applied to B-cell differentiation data, SPD recovered the correct order of stages of normal B-cell differentiation and the linkage between preB-ALL tumor cells with their cell origin preB. When applied to mouse embryonic stem cell differentiation data, SPD uncovered a landscape of ESC differentiation into various lineages and genes that represent both generic and lineage specific processes. When applied to a prostate cancer microarray dataset, SPD identified gene modules that reflect a progression consistent with disease stages. SPD may be best viewed as a novel tool for synthesizing biological hypotheses because it provides a likely biological progression underlying a microarray dataset and, perhaps more importantly, the candidate genes that regulate that progression.

## Author Summary

We present a novel computational approach, Sample Progression Discovery (SPD), to discover biological progression underlying a microarray dataset. In contrast to the majority of microarray data analysis methods which identify differences between sample groups (normal vs. cancer, treated vs. control), SPD aims to identify an underlying progression among individual samples, both within and across sample groups. We validated SPD's ability to discover biological progression using datasets of cell cycle, B-cell differentiation, and mouse embryonic stem cell differentiation. We view SPD as a hypothesis generation tool when applied to datasets where the progression is unclear. For example, when applied to a microarray dataset of cancer samples, SPD assumes that the cancer samples collected from individual patients represent different stages during an intrinsic progression underlying cancer development. The inferred relationship among the samples may therefore indicate a trajectory or hierarchy of cancer progression, which serves as a hypothesis to be tested. SPD is not limited to microarray data analysis, and can be applied to a variety of high-dimensional datasets. We implemented SPD using MATLAB graphical user interface, which is available at http://icbp.stanford.edu/software/SPD/.

## Introduction

Biological processes of development, differentiation and aging are increasingly being described by the temporal ordering of highly orchestrated transcriptional programs [1]. When such processes are analyzed with gene expression microarrays at specified time points, a variety of computational methods are available to identify which genes vary and how they vary across part or all the time points [2], [3], [4], [5], [6]. However, when microarray samples of a biological process are available but their ordering is not known, fewer methods are available to recover the correct ordering, especially when the underlying process contains branchpoints, as occurs in the differentiation from hematopoietic stem cells to myeloid and lymphoid lineages. We present a novel method, referred to as Sample Progression Discovery (SPD), to discover the progression among microarray samples, even if the progression contains branchpoints. In addition, SPD simultaneously identifies genes that define the progression. SPD can be used to generate biological hypotheses about a progressive relationship among samples, and the genes that serve as key candidate regulators of the underlying process.

Recovery of an ordering among unordered objects has been studied in a variety of contexts. In computer vision, images taken from random viewpoints and angles were ordered for the purpose of multi-view matching [7], where the ordering was based on predefined features that are invariant to different viewpoints. In genetics, spanning trees were applied to reconstruct genetic linkage maps [8], which was an ordering of genetic markers. Using gene expression data of a small set of preselected genes, phylogenetic trees were constructed to study cancer progression among microarray cancer samples [9], [10]. Microarray samples were also ordered by a traveling salesman path from combinatorial optimization theory, but feature selection was not discussed [11], [12]. Although these methods proved useful in the recovery of an ordering from unordered objects, their direct applications cannot address the challenges of extracting progression and differentiation hierarchy from microarray gene expression data. Algorithms in [7], [11], [12] assume linear ordering of unordered objects, and therefore are not able to reveal potential branchpoints. Furthermore, most existing methods order samples based on carefully designed or preselected features. However, in microarray gene expression data, meaningful gene features are usually not known *a priori*. One important aspect of SPD is its feature selection ability. Assuming the underlying progression can be reflected by gradual expression changes of subsets of genes, SPD selects genes whose gradual changes support a common progression, and hypothesizes that the common progression is biologically meaningful.

**Figure 1:**
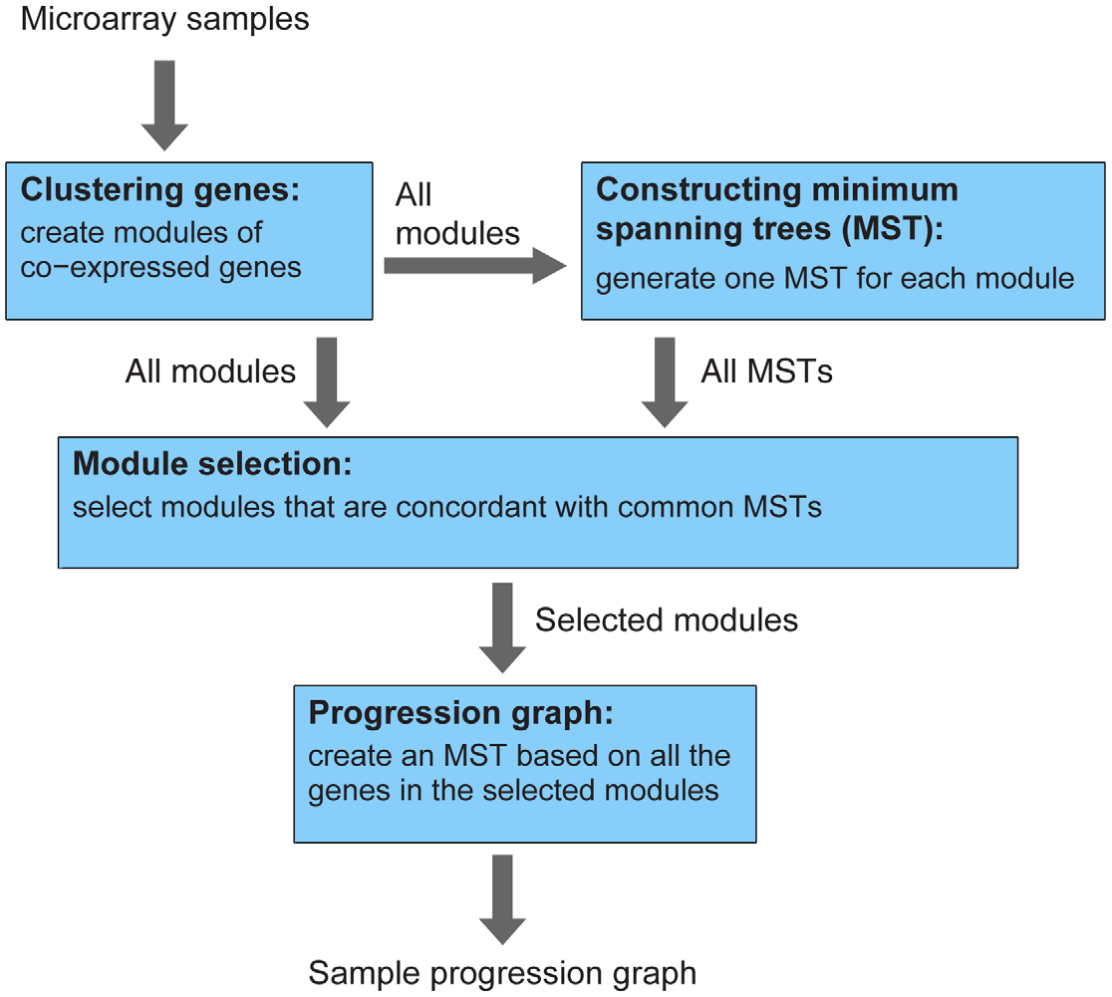
Sample Progression Discovery (SPD) framework.

The SPD framework, as depicted in Figure 1, discovers biological progression from gene expression microarray data using four steps: (1) cluster genes into modules of co-expressed genes, (2) construct minimum spanning tree (MST) for each module, (3) select modules that supports common MSTs, and (4) reconstruct an overall MST based on all the genes of all the selected modules. Gene clustering is needed to reduce the number of gene expression patterns to be tested. We introduce an iterative consensus k-means algorithm to derive coherent gene modules, where genes within each module are highly co-expressed. For each gene module, a minimum spanning tree (MST) is constructed [13], where the nodes of the MST are the microarray samples and the edges are weighted by the distance between samples' gene expression profiles. Because a MST connects samples using the minimum number of edges and minimum total edge weights, it tends to connect samples that are more similar to each other. Therefore, we use the MST to describe the progression among the samples, defined by the gradual change of the corresponding gene module. The progression is not necessarily linear, and can contain branching points.

SPD then performs feature selection by evaluating the statistical concordance between each gene module and each MST. We define a measure of “progression similarity” between two modules as the number of MSTs that are concordant with both of the two modules. If two modules are concordant with the same MST, these two modules share progression similarity, because their gradual changes support a common progression order among the microarray samples. A noteworthy point here is that modules that are concordant with the same MST are not necessarily correlated with each other; hence SPD is able to identify similarities that may be missed by correlation or regression-based analyses [14], [15], [16]. If there exist multiple modules that are concordant with a common set of MSTs, these modules support a common progression, which is likely to be biologically meaningful. SPD selects modules that share progression similarity, and constructs an overall MST based on all the genes within the selected modules. The overall MST and the selected modules serve as the basis for generating hypotheses of the underlying biological process and its regulators.

To demonstrate the potential of SPD to reveal biological processes underlying microarray samples, we applied it to a variety of microarray datasets, each of which was associated with a known biological progression. In each case, the known progression was hidden from SPD, and was used to validate the progression discovered by SPD. First, SPD was applied to a time series microarray dataset of the cell cycle. SPD successfully recovered the correct time order of the samples and identified many genes that have been associated with the cell cycle. When applied to B-cell differentiation data, SPD recovered the correct order of different stages of normal B-cell differentiation, and identified the linkage between preB-ALL tumors and their preB cell of origin. SPD was applied to a mouse embryonic stem cell differentiation dataset, where SPD uncovered a landscape of ESC differentiation into various lineages and genes that represent both generic and lineage specific processes. When applied to a prostate cancer microarray dataset, SPD identified gene modules that reflect a progression consistent with disease stages. All of these applications of SPD are presented in the following sections and, collectively, show that SPD may be best viewed as a novel tool for synthesizing biological hypotheses, because it provides a likely biological progression across microarray samples and, perhaps more importantly, the candidate genes that regulate the progression. We implemented SPD using MATLAB graphical user interface. Our software is available at http://icbp.stanford.edu/software/SPD/.

## Results

### SPD recovers temporal information of cell cycle time series data

Microarray time series data of the cell cycle were used to evaluate the performance of SPD. Information on the temporal sample order and cell-cycle regulated genes were not provided to SPD. We hypothesized that SPD would recover the underlying biological progression (in this case, the cell cycle) and identify the genes associated with that progression. Five cell cycle time series gene expression datasets in [17] were independently analyzed by SPD. Here we present the SPD analysis on the series with the largest number of samples. SPD analysis of the other time series can be found in Supplement Text S1.

**Figure 2:**
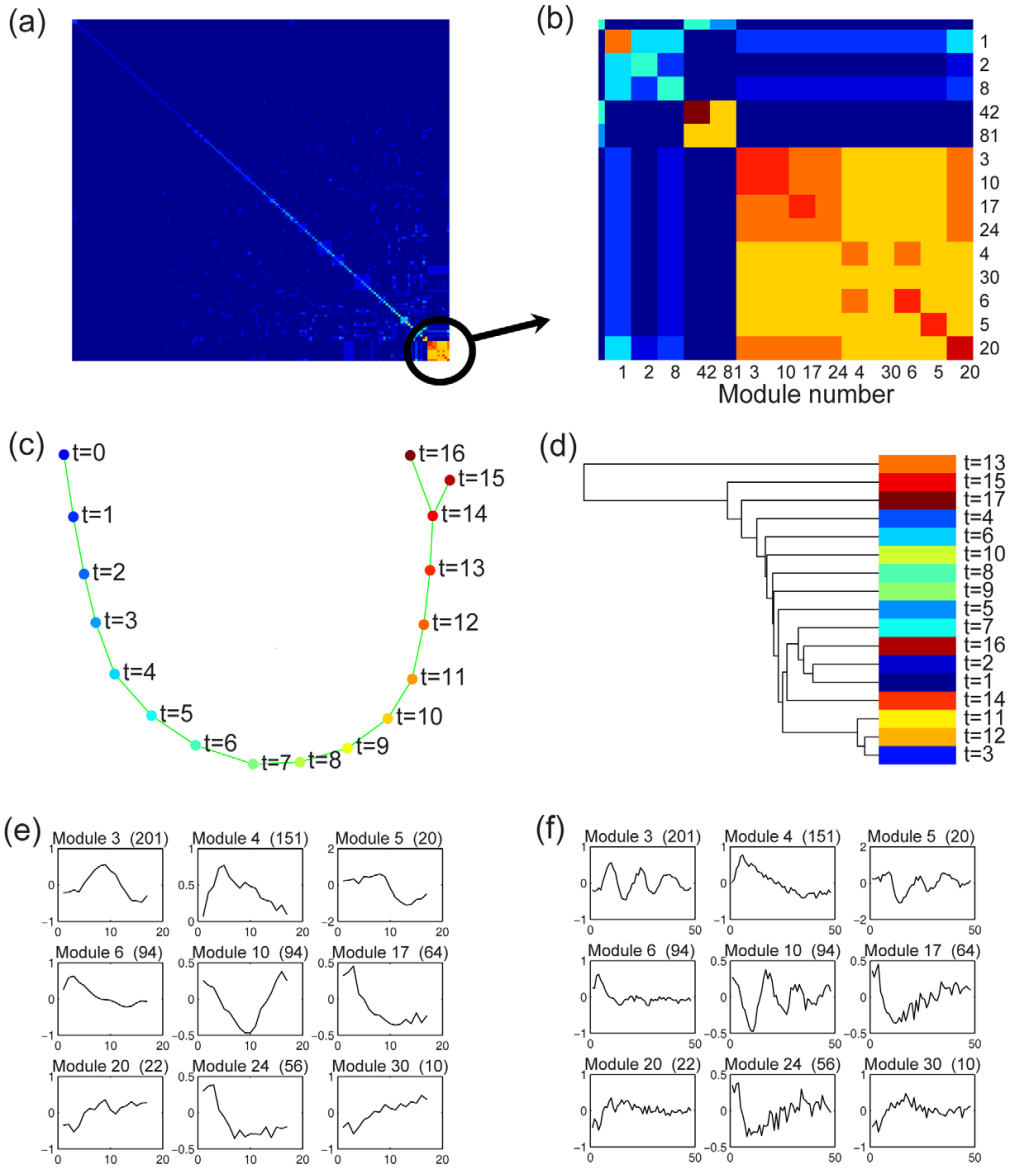
SPD applied to a cell cycle gene expression dataset. (a) Based on an expression matrix of 3196 genes for 17 unordered samples from one cell cycle, SPD derived 154 modules and a progression similarity matrix between them. (b) Zoomed-in view of the progression similarity matrix highlights nine modules that are similar in terms of progression. (c) SPD constructed an overall MST to describe the common progression supported by the nine modules, showing a near perfect reconstruction of the correct time order. (d) Hierarchical clustering analysis did not recover the correct time order. In (c) and (d), Samples were color-coded according to the time points (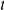, hours), when the samples were taken. Blue corresponds to earlier time points; red corresponds to later time points. (e) The average expressions of the nine modules across the 17 time points show that, some of the nine modules were uncorrelated, i.e., modules 10 and 30, but SPD identified them as similar in terms of progression. (f) The mean expressions of the nine modules across all three cell cycles. The number in the parentheses above each plot is the number of genes in the corresponding module.

The input of SPD was a gene expression data matrix of 3196 high variance genes across 17 unordered samples from only one cell cycle. The SPD analysis was deliberately limited to samples in one cell cycle to avoid the possibility that SPD would order the samples using the cyclic behavior of cell-cycle regulated genes that can be easily observed across multiple cell cycles. SPD clustered the 3196 high variance genes into 154 modules of co-expressed genes, using an iterative consensus k-means approach (see Methods). One MST was constructed for each module. Each MST represented a possible progression order of the samples based on the expression of its corresponding module. Then, a progression similarity matrix was constructed to quantify the similarity between pairs of modules. The (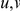) element of the progression similarity matrix was defined as the number of MSTs concordant with both modules 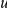 and 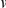. (see Methods). The progression similarity matrix is shown in Figure 2(a), and a magnified portion is shown in Figure 2(b) to highlight nine modules (3, 10, 17, 24, 4, 30, 6, 5 and 20) that are regarded as similar in terms of progression. In the last step of SPD, the nine modules with the highest progression similarity were combined to construct an overall MST. The overall MST was visualized using high-dimensional embedding, shown in Figure 2(c), and revealed a near perfect restoration of the sample order. Interestingly, when we examined the MSTs constructed from each of the nine modules, we did not recover the correct order because we were essentially projecting the progression into a lower dimensional space. To demonstrate the value of the overall MST versus the individual-module MSTs for restoring the sample order, we applied a distance metric called topological overlap measure (TOM) [18] to evaluate the distance between the MSTs and the true sample order. Table 1 shows that the overall MST based on combining the nine modules (the first row) produced a more accurate sample order than the MSTs derived from the individual modules.

**Table 1:** The topological overlap measure between extracted sample progression patterns and the true time order.

	TOM network distance
SPD	4.33
Module 3 MST	17.33
Module 4 MST	19.33
Module 5 MST	24.00
Module 6 MST	18.33
Module 10 MST	20.17
Module 17 MST	35.67
Module 20 MST	45.57
Module 24 MST	29.33
Module 30 MST	26.00
single linkage HC	42.50

Next, we compared SPD to the commonly used hierarchical clustering analysis of the dataset described above. After all, a MST can be regarded as a hierarchical clustering tree with single linkage [19]. The main difference between hierarchical clustering and the SPD framework is that SPD selects gene modules that share progression similarity, and reconstructs an overall MST based on the selected modules. To illustrate the significance of SPD's feature selection ability, we performed single linkage hierarchical clustering based on all the 3196 genes, which is equivalent to constructing a MST based on all these genes. The resulting dendrogram did not recover the correct sample order, as shown in Figure 2(d). Moreover, the TOM distance between the hierarchical clustering tree and the true sample order was much larger than that from SPD, as shown in Table 1 (last row). This analysis demonstrates the importance of SPD's feature selection ability.

To evaluate the robustness of SPD, we performed bootstrap analysis on the cell cycle microarray dataset. In each of the 100 bootstrap iterations, 90% of the 3196 genes were randomly selected. SPD was applied to each bootstrapped dataset separately. In each bootstrapped dataset, the clustering step might generate different gene modules that lead to different progression-related modules and a different overall MST. However, the overall MSTs were consistent across the bootstrapped datasets. The TOM distance was used to evaluate the distance between the 100 SPD results and the true sample order. The mean TOM distance was 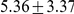. The standard deviation of the TOM distance appeared to be comparable to the mean due to the statistical properties of TOM. To evaluate the statistical significance of this result, we performed random permutation analysis. We generated 1000 random MSTs, and computed the TOM distance between random MSTs and the true sample order. The mean of the random TOM distance was 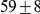, which is substantially larger than the TOM distances obtained in the bootstrap analysis, indicating the robustness of SPD. In addition, we examined the diameters of the random MSTs, where the diameter is defined as the number of edges in the shortest path between the most distantly separated pair of nodes. The mean diameter of a random 17-node MST was 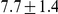. The diameter of the SPD result in Figure 2(c) was 15. The probability of obtaining such a large diameter by chance was low, which implied that the SPD result was statistically significant.

The mean expression profiles of the nine modules are shown in Figure 2(e). Some of these modules are uncorrelated (i.e., modules 10 and 30 have a correlation coefficient of −0.06), but SPD identified them as similar in terms of progression. Figure 2(f) shows the mean expression profiles of the nine modules across all three cell cycles that were provided in the original dataset. Here, we can observe that some of the identified modules are cyclic. Gene sets in the Molecular Signatures Database (MSigDB) [20] were used to annotate the identified gene modules (see Supplement Text S1). As expected, these modules included many genes that have been associated previously with the cell cycle. For example, module 10 was highly enriched (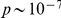, hypergeometric test for gene set enrichment) for genes that are targets of the E2F cell cycle transcription factor family. A likely explanation for the presence of the acyclic modules is that they represent the experimental perturbation that initially synchronized the cells. In the cell cycle microarray experiments, the measured population of cells were first synchronized, and then released. This initializing synchronization condition is a cellular perturbation that may take several cell cycles to decay away.

### SPD recovers stages of B-cell differentiation

We applied SPD to a B-cell differentiation dataset [21], in which 9365 genes were measured for 44 samples across 5 normal differentiation stages and 1 malignant stage: 7 hematopoietic stem cells (HSC), 7 common lymphoid progenitors (CLP), 7 proB cells, 7 preB cells, 7 Immature B cells (IM), 5 more terminally differentiated B cells (1 naive B cell, 1 centroblast CB, 1 centrocyte CC, 1 memory B cell, 1 CD19+ cell), and 4 preB-ALL cancer samples. Without providing SPD any information on the known differentiation stages of the sample, we tested whether SPD could recover the progression underlying this dataset, which is known to be: HSC, CLP, proB, preB, IM, naiveB/CB/CC/memoryB/CD19+. Another objective was to determine whether the preB-ALL would be grouped near its preB cell origin.

**Figure 3:**
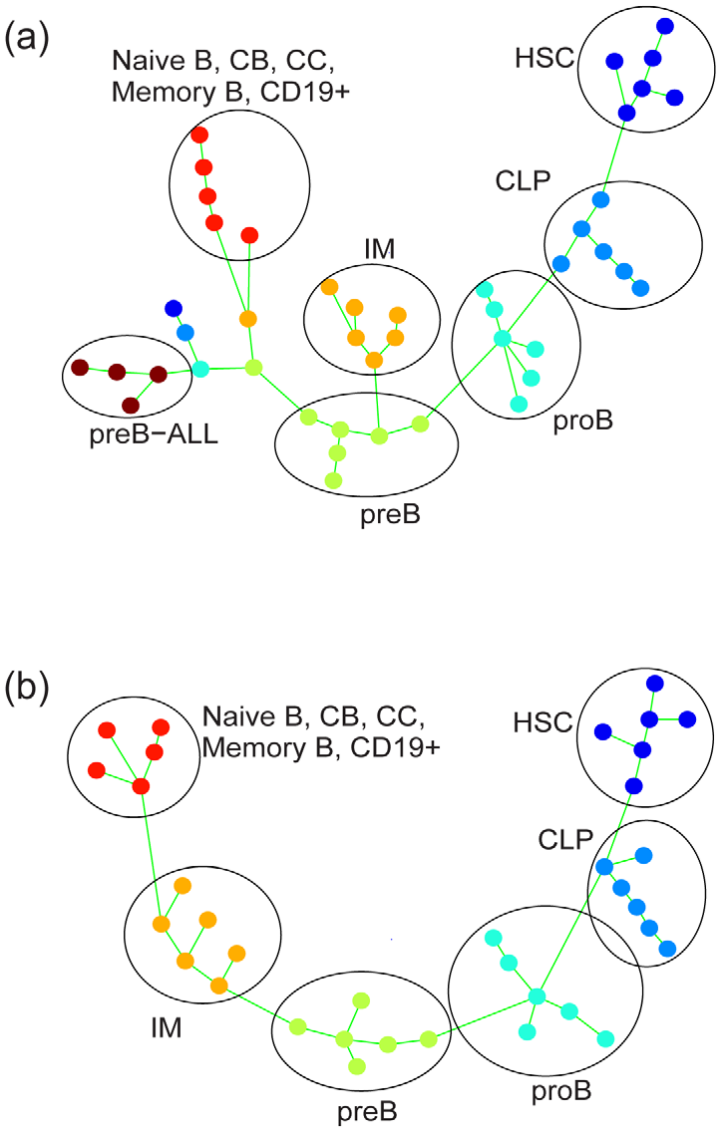
SPD applied to gene expression data of B-cell differentiation. (a) Analysis based on all samples in this dataset. (b) Analysis of normal samples, with cancer samples and outliers next to cancer samples removed. Samples are color-coded according to their classification: HSC (violet), CLP (blue), proB (light blue), preB (green), immature (yellow), naiveB/CB/CC/memoryB/CD19+ (red), preB-ALL (brown). Circles are added to highlight each class of samples.

SPD selected ten gene modules (composed of 2388 genes in total) which supported a common progression. Based on these modules, an overall MST was constructed to describe the underlying progression. After obtaining the overall MST, samples were color-coded according to their known classifications, as shown in Figure 3(a). The identified progression was consistent with the known stages of normal B-cell differentiation, except for a missing link between immature B cells and the next more differentiated B cells (naiveB/CB/CC/memoryB/CD19+). The link between preB-ALL cancer samples and their cell origin (normal preB cells) was identified.

The link between immature B cells and more differentiated B cells was missing, partly because MSTs do not allow for cycles. We hypothesized that if we removed the cancer samples and the outliers that are grouped next to the cancer samples, the missing link would be restored. To test this, we removed the cancer samples and the outliers, and performed SPD analysis again. The resulting MST was consistent with the stages of normal B-cell differentiation, as shown in Figure 3(b).

Annotations of the selected modules can be found in Supplement Text S1. Some modules contained genes that relate to B-cell differentiation but are generic in their function. Examples include proliferation genes (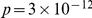, hypergeometric test), which are highly expressed in germinal center B-cells that are undergoing rapid expansion, but down-regulated at other stages. SPD also recovered modules of genes that are specific to B-cell differentiation. These were enriched in genes that are known markers of, or mechanistically related to, B-cell differentiation such as CD19, CD20, CD79 (B-cell receptor), and the master transcription factors PAX5 and SP140. We also observed enrichment (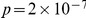) for genes in the B-cell receptor (BCR) pathway, which is the key signaling pathway governing the maturation of B cells.

### SPD reveals landscape of embryonic stem cell differentiation

The two examples in the previous subsections show that SPD is able to recover non-branching progression patterns. In this subsection, we demonstrate SPD's ability to recover branched progression patterns, using an embryonic stem cell differentiation dataset. Pluripotent embryonic stem cells (ESCs) are capable of differentiating into all cellular lineages in the development of a mature organism. We applied SPD to a dataset of 44 samples of mouse ESCs and their progeny which had been induced to differentiate into several lineages by specific interventions, as well as several differentiated cell types. The interventions included knockdown of Pou5f1/Oct4 (leading to differentiation to trophoblasts), induction of GATA6 (differentiation to endoderm lineage), treatment with N2B27 medium (differentiation to neural lineages), and all-trans retinoic acid (RA) induction of embryonic carcinoma cells to cause differentiation [22]. The data included time series along each lineage of cells.

**Figure 4:**
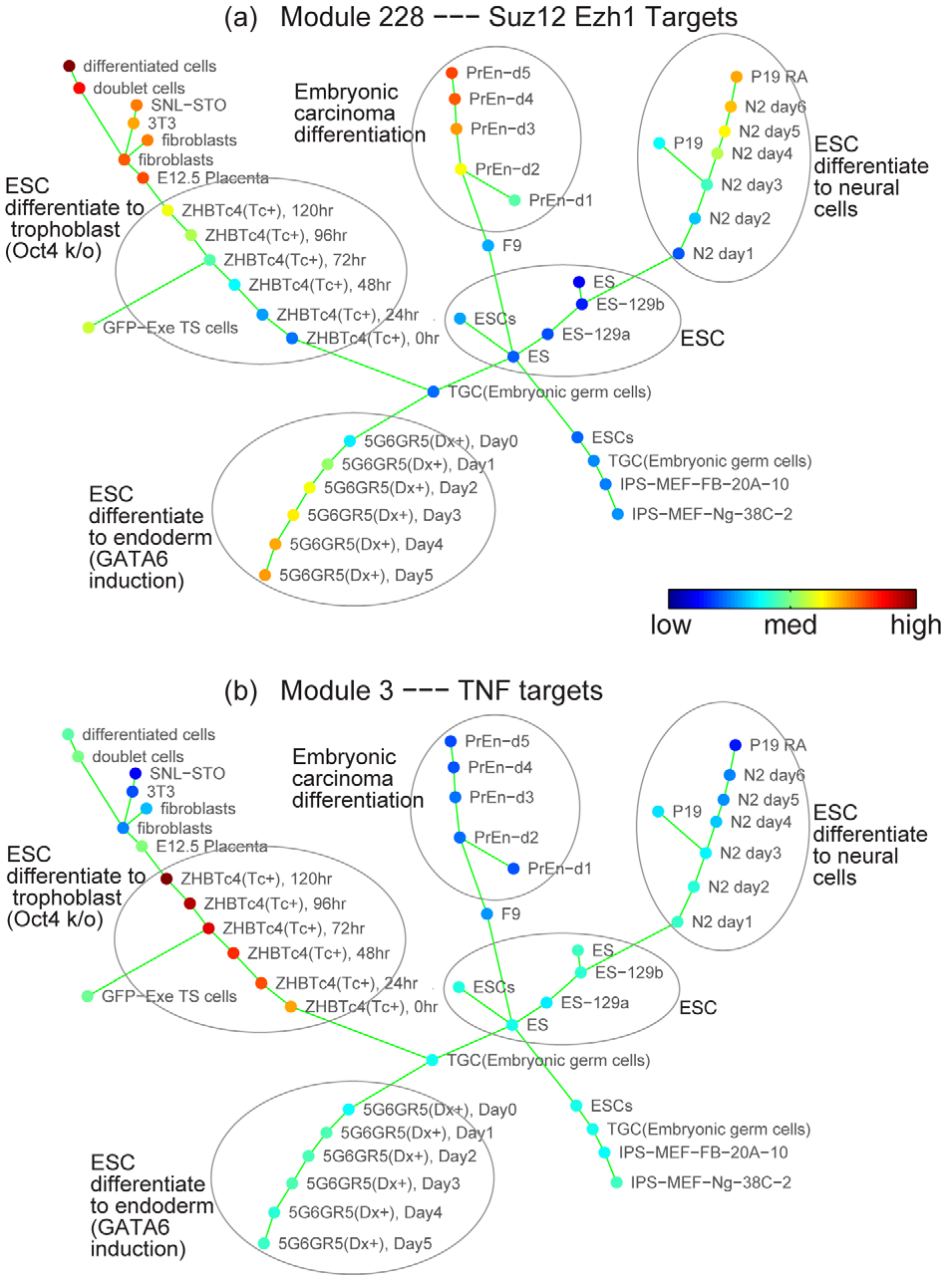
SPD applied to mouse embryonic stem cell differentiation data. SPD revealed a landscape of mouse embryonic stem cell differentiation, where samples were perfectly ordered in time, with progressively later stages of differentiating cells radiating outwards from a core cluster of ESC samples. Circles were added to highlight each lineage. Nodes were color-coded by the expression level of a gene module (blue means low expression; green/yellow means medium; red means high expression). Module 228 was progressively induced in all differentiating lineages, and was enriched for Suz12 and Ezh1 targets. Module 3, enriched by TNF targets, was highly specifically regulated along one lineage, the trophoblast.

When SPD was applied to this dataset, information on the interventions and the temporal order of the samples were hidden from the algorithm. SPD identified 35 modules that supported a common progression, which revealed a landscape of ESC differentiation into the various lineages. Remarkably, samples were perfectly ordered in time, with progressively later stages of differentiating cells radiating outwards from a core cluster of ESC samples, as shown in Figure 4. A subset of induced pluripotent (iPS) cells clustered as a group, in close proximity to ESC samples. Trophoblast stem cells grouped with the trophoblast differentiation lineage, while stromal and fibroblast cell lines were correctly clustered with mature fibroblasts. The identified modules provided a fine-scale view of expression changes along each lineage. The identified modules included ones which changed in a similar fashion during differentiation of all cell types from ESCs, as well as ones that were uniquely associated with specific lineages. We annotated modules by comparison to known gene sets, and by examining the relationship between their constituent genes using Ingenuity Pathways Analysis (IPA). Annotation results are available in Supplement Text S1.

Module 228 was progressively induced in all differentiating lineages, as shown in Figure 4, and was enriched for genes that are targets of Suz12 and Ezh1. The latter are members of the Polycomb complex that functions in maintaining self-renewal in ESCs. Thus induction of this module is consistent with a general loss of self-renewal potential in specialized cell types. Similarly, modules 54 and 55 (enriched for Myc targets and genes involved in Oct4 maintenance of pluripotency) were both down-regulated in each differentiating branch, but at differing rates with respect to each other, with the strongest muting of expression occurring along the trophoblast lineage. One module (329) was highly enriched for genes that share a common pattern of histone H3K27 methylation, and that are targets of the Ezh2/Polycomb complex. Notably these genes were progressively down-regulated in all branches except the neural lineage. This suggests particular subsets of Polycomb targets that are regulated in a tissue-specific manner. In the opposite direction, module 65 genes were strongly induced in trophoblast differentiation, and more modestly in the other branches. This module contained numerous genes that are induced by shRNA knockdown of the pluripotency factor Sox2, as well as apoptosis-related genes. Intriguingly, this module included many genes involved in integrin signaling and endocytosis signaling. Thus its strong induction in differentiating trophoblasts (which are involved in placental implantation of the embryo) is consistent with their critical invasive properties, and the SPD result identifies genes that may be implicated in this phenotype.

Two modules (3 and 123) were highly specifically regulated along the trophoblast differentiation branch. IPA analysis of module 3 indicated that this cluster of genes was highly enriched with targets of tumor necrosis factor (TNF). This is concordant with the fact that over-expression of TNFa induces differentiation of ESC to trophoblasts. In the dataset analyzed with SPD, trophoblast differentiation was induced by down-regulation of Oct4. The overlap with TNF targets suggests that these two mechanisms of induction share commonalities in the gene expression changes involved in generation of trophoblasts from ESC. Given the master-regulatory role of Oct4 in maintaining pluripotency, one hypothesis is that induction of TNF effects downstream changes in the Oct4 network, while at the same time producing changes in transcription that lead specifically to production of trophoblasts. Module 123 was annotated as associated with cell motility genes. Again, this is consistent with the invasive character of trophoblasts, and suggests genes that are involved in mediating this behavior.

In summary, SPD perfectly recapitulates the lineages leading to differentiated cell types generated by targeted manipulations of ESCs. The differentiation landscape identified by SPD shows underlying progressive changes in gene expression that represent both generic processes as well as ones specific to particular lineages. The genes that constitute the modules supporting the differentiation tree represent targets for further investigation as to their role in organism development.

### SPD reveals stages of prostate cancer progression

We applied SPD to a prostate cancer microarray dataset [23]. This dataset contains a total of 163 patient samples, including tissue of normal prostate from organ donors, normal prostate tissue adjacent to the prostate tumor (NAP), primary prostate tumor samples, and metastatic samples. When SPD was applied to this dataset, the clinical information on the samples were hidden from the algorithm.

**Figure 5:**
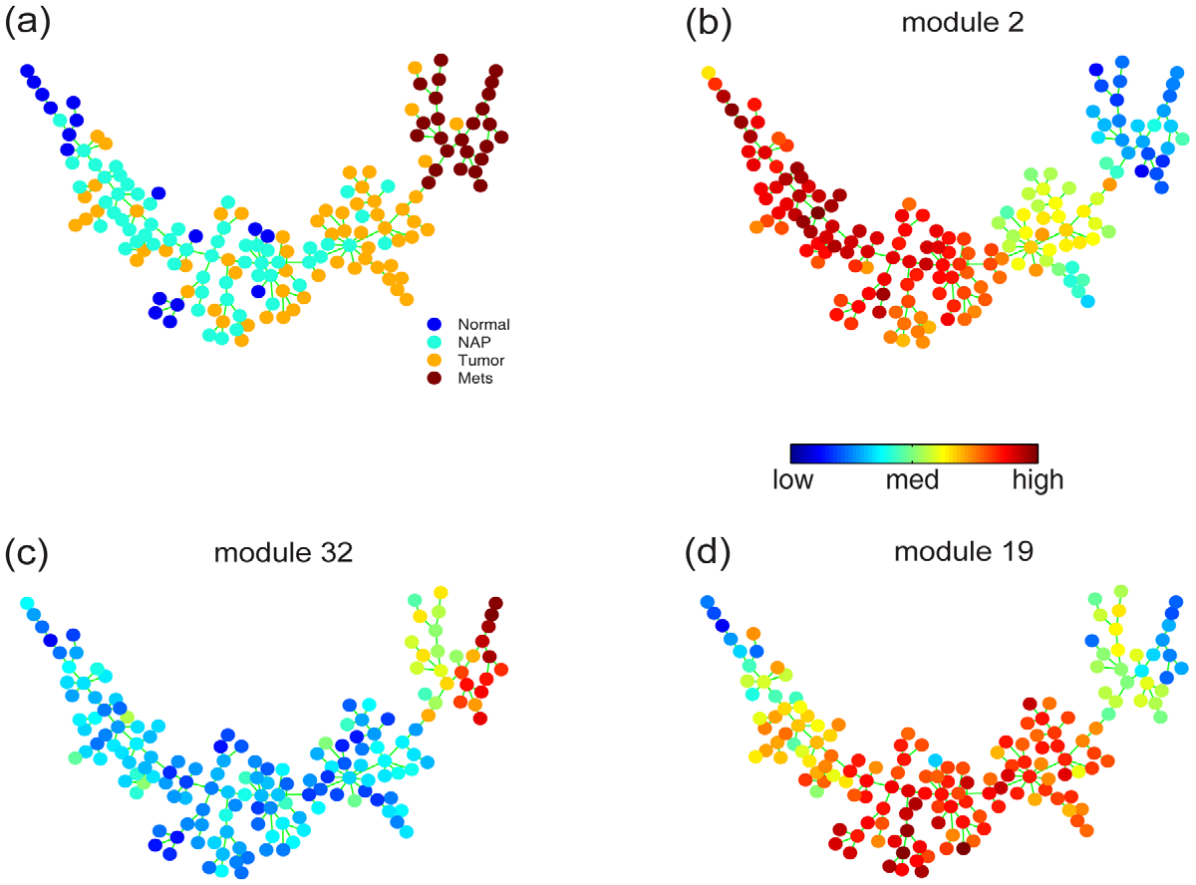
SPD applied to a prostate cancer dataset and derived a tree structure that describes the underlying progression. (a) Nodes were color-coded according to their classification: normal, normal adjacent to tumor (NAP), tumor, metastatic samples (Mets). In (b), (c) and (d), nodes were color-coded using the average expression of modules 2, 32 and 19, respectively, in order to show how the expression of these modules gradually change during the progression.

In this dataset, the average correlation between genes was small, consequently, SPD generated modules that contained a small number of genes. We excluded modules that contained less than 5 genes, leaving 46 coherent modules for subsequent analysis. SPD selected 12 modules (487 genes in total) with high progression similarity and derived the tree structure shown in Figure 5(a). Normal and metastatic samples were enriched at the left and right ends of the tree. SPD produced a mixture of NAP and tumor samples in the middle of the tree. A larger fraction of NAP samples were closer to normal samples, and tumor samples were closer to the metastatic samples. The mix of NAP and tumor samples reflects possible field effect [23], which suggested that normal tissue adjacent to primary tumor is more similar to the primary tumor than it is to normal tissues. The general trend in Figure 5(a) reflected a progression consistent with disease stages. In addition, we observed details that we did not expect: a few normal samples were mixed with tumor samples; and the metastatic samples appeared to form two branches.

To interpret the tree, we color-coded the nodes using the average gene expression of each of the 12 modules, and observed three expression patterns. Representative modules of the three patterns are shown in Figure 5(b), (c) and (d). Color-codes of other modules are available in Supplement Text S1. Module 2 and three other modules are gradually down-regulated from normal to tumor and metastatic samples, whereas module 32 and two other modules are gradually up-regulated. Interestingly, we observed that the expression of module 19 and four other modules first increase from normal to tumor and then gradually decrease in metastatic samples. Several modules show clear difference between the two branches in the upper right corner, i.e. Figures 5(c), (d) and several modules shown in Supplement Text S1. The expressions of these modules indicate that the metastatic samples can be further divided into two subtypes.

We used Gene Set Enrichment Analysis to annotate the modules that are up-regulated in primary tumor while down-regulated in both normal and metastatic samples. We noticed that these modules overlapped with genes involved in metastasis in several epithelial cancers (not just prostate studies). They may reflect general processes underlying the epithelial-mesenchymal transition and cell migration. Of note, one of the genes in this module is CDH3, a member of the cadherin family that interacts with CDH1. Targeted down-regulation of cadherins by RNA interference has been demonstrated to induce cell migration. However, up-regulation from normal to primary tumors followed by down-regulation in metastases has not been commented upon previously. We also applied IPA to the genes that comprised these modules. The most significant interaction network centered around genes involved in androgen and estrogen signaling, and influenced by beta-estradiol. Although estradiol is the predominant sex hormone in females, it is also produced in males as a metabolic product of testosterone. Androgen signaling generally has a pro-survival effect in prostate cancers. Thus one possible interpretation of the SPD result is that it reflects the fact that in primary tumors, androgen signaling up-regulation confers a selective advantage in the natural history of the tumor; but that some metastases develop androgen-independence. A priori, from gene expression profiles, it is unknown which metastases are androgen-independent; hence SPD may be identifying both androgen-independent samples, together with the genes whose changes in expression drive the phenomenon.

## Discussion

SPD is a new approach to infer progression among microarray samples and identify genes that drive the progression. SPD represents a new class of machine learning algorithms that has not been extensively applied to microarray analysis. The more common machine learning algorithms that have been used to analyze microarray data include unsupervised clustering [19], [24], supervised classification [25], [26], [27], [28], and statistical tests for differential expression [20], [29]. Although these algorithms are quite different from each other, they share a similar goal, which is to identify differences between different sample groups, i.e. normal vs. cancer. When applied to study a progressive biological process, these methods essentially bin the process into stages and identify differences between sample groups from consecutive stages of the progression. SPD differs significantly in this regard. Instead of assuming that samples in the same group are similar and focusing on the differences between groups, SPD treats individual samples as different points along an unknown biological progression, thus has the potential to discover how samples progress both within and across groups. As mentioned earlier, recovery of an ordering from unordered samples has been studied in several fields, computer vision, [7], genetic linkage analysis [8], [9], [10], microarray time series [11], [12]. However, due to lack of ways to automatically select meaningful features, the direct application of these approaches cannot address the challenges of extracting unknown progression from microarray data. In contrast, SPD is unique in its ability to simultaneously identify both the progression relationship among samples and the genes that are associated with the progression, without prior knowledge or manual gene selection.

SPD shares some similarities with bi-clustering, since both methods attempt to simultaneously organize genes and samples. However, the results of SPD and bi-clustering are quite different from each other. Bi-clustering organizes genes into clusters, and each gene cluster stratifies samples in a potentially different way. In contrast, SPD has a module selection step which selects the gene modules that are similar in terms of progression. The selected modules support a common progression pattern among the samples, defined by a single overall MST which is constructed based on all the genes in the selected modules.

In SPD, we propose a new similarity measure, namely the progression similarity. This measure evaluates the similarity between gene modules based on whether they are concordant with common progression patterns represented by MSTs. In contrast to correlation and regression-based methods [14], [15], [16] where the expression profiles of gene modules are directly compared with each other, SPD evaluates progression similarities between gene modules via MSTs. We have shown that modules that are similar in SPD do not necessarily correlate with each other; this demonstrates that SPD is able to identify similarities that correlation and regression-based analyses may miss.

As demonstrated in the analysis of the cell cycle time series and B-cell differentiation microarray data, SPD is able to discover the biological progression underlying a microarray dataset, while simultaneously selecting the genes that are known to be central to this progression. When applied to these datasets, SPD was not provided the information on the known ordering of the samples, and instead derived the ordering in an unsupervised fashion. Because the SPD-derived ordering is consistent with the time order of the samples, time represents the strongest progression signal, and the gradual shifts of the identified gene modules are associated with the time series experiment. Enrichment of transcription factors or pathways in the identified modules may be hypothesized as key drivers of the progression, and subject to further experimental validation. If the SPD-derived ordering were different from the time order, the strongest progression signal would be some factor other than time, which hints at other sources of variations present in the time series data.

We view SPD as a hypothesis synthesis tool that may have greatest utility when applied to a microarray dataset where the underlying biological progression is unknown. For example, when applied to cancer samples, SPD assumes that there is an intrinsic progression underlying cancer development, and that the cancer samples collected from individual patients represent different stages in this progression. The inferred progression relationship among the cancer samples may therefore indicate a trajectory or hierarchy of cancer progression. Under this assumption, SPD extracts the progression among cancer samples and gene modules whose gradual shifts are associated with the progression, as demonstrated on human prostate cancer samples. The identified progression and gene modules form hypotheses to be validated. SPD is not limited to microarray data analysis and can be applied to a variety of high-dimensional datasets, including genomic, proteomic and image-based data.

## Methods

### Iterative consensus clustering

Gene clustering reduces data dimension and noise. It is well known that gene clustering is a difficult optimization problem with many local minimums, and most clustering algorithms lack consistency and reproducibility across multiple runs [30]. We propose an iterative consensus k-means algorithm to derive consistent coherent gene modules. Our algorithm is an iterative divisive hierarchical clustering procedure. In every iteration, each gene module from the previous iteration is divided into two modules, until our stopping criterion is met. Details of the algorithm are as follows.

Given an 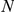 by 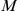 gene expression data matrix, we perform the k-means algorithm 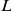 times, with random initialization, to cluster the 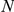 genes into k = 2 clusters. Clustering results are arranged into an 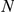 by 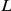 matrix, where the 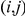 element is the cluster assignment of gene 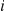 in the 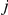
'th run of k-means. In order to draw the consensus of the 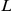 runs of k-means, we apply k-means again based on the 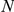 by 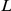 matrix, the collection of clustering results of the 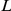 runs, to divide genes into two clusters. For each of the two clusters, the coherence is computed as the average Pearson correlation between each gene in the cluster and the cluster mean. If the coherence of a cluster is higher than a pre-specified threshold 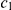, this cluster is considered to be a coherent gene module. Otherwise, this cluster is further partitioned by iterating the algorithm. After the iterative process ends, we examine the resulting coherent modules pairwisely. If the Pearson correlation of two modules' centers is higher than a pre-specified threshold 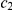, these two modules are merged. This step iterates until no module-pair shares correlation higher than 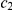. The stopping criterion of cluster coherence guarantees that all resulting modules satisfy the pre-specified coherence threshold 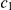. Modules that share correlation higher than 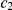 are merged, so that the resulting gene modules are not highly correlated with each other. We typically set the algorithm parameters to the following values: 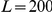, 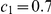, 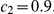


The purpose of our consensus k-means algorithm is to derive coherent modules that are not highly correlated with each other. Other clustering algorithms that achieve qualitatively similar performance can be adopted as the clustering component of SPD. When dealing with microarray gene expression data, without any prior knowledge of gene modules and the underlying progression, we find it helpful to cluster co-expressed genes into modules for the purpose of dimension reduction. On the other hand, if we have prior knowledge of predefined gene sets that describe pathways whose progression similarities are of interest, we can use these genes sets to supplement or replace the clustering results.

### Constructing minimum spanning tree

SPD constructs minimum spanning trees (MSTs) based on expression data of subsets of genes, i.e. gene modules. A MST is an acyclic graph that connects all the samples using minimum total edge weights. The weight on the edge that connects samples 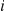 and 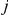 is defined as the Euclidean distance between the gene expression of samples 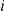 and 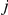. We use Boruvka's algorithm [31] to construct one MST based on each gene module. Briefly, we begin with a disjoint graph with no edges, where each sample is one disjoint component, and then iteratively add edges. In each iteration, we randomly pick one of the smallest components, calculate its single linkage Euclidean distances to all other components, and add an edge that corresponds to the smallest single linkage distance. This process iterates until all samples are connected.

Since the MST connects all the nodes using minimum total edge weights, it tends to connect samples that are more similar to each other. If we start from one sample and move along the edges of the MST, we will observe a gradual change of gene expression. Therefore, the MST reflects the progression among samples, defined by the gradual change of the set of genes based on which the MST is constructed.

### Statistical concordance between modules and trees

The key step of SPD is the comparison between the expression of gene modules and trees constructed from other modules. Given the expression data of a gene module in 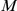 samples, we define an 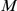 by 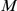 distance matrix 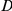, where 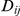 is the Euclidean distance between the gene expression profiles of samples 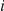 and 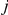. Similarly, a tree structure can also be summarized in a matrix form, which is the adjacency matrix 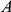, where 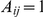 if samples 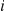 and 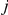 are directly connected in the tree; otherwise 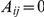. In SPD, we define the concordance between a gene module and a tree as the concordance between the distance matrix 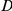 and the adjacency matrix 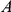.

Typically, the statistical concordance between 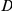 and 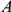 includes two aspects: (1) the distance between connected samples should be small, and (2) the distance between not-connected samples should be relatively larger [32]. In our analysis, we only focus on the former aspect. Our rationale is that we want to model progressions where the gene expression first drifts away from an initial state and then comes back. One such example is the cell cycle. We define the statistical concordance between a distance matrix 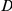 and an adjacency matrix 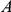 as 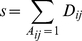
(1)


The meaning of 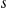 is the total edge weights jointly defined by the gene module and the tree. If 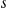 is small, the gene module 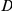 and tree structure 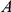 are concordant. Large 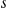 implies that the gene module 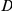 and tree 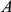 are not concordant. In order to derive the 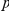-value of 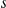, we perform random permutations. We randomly permute the columns of the expression data, which is equivalent to reshuffling the rows and columns of the distance matrix 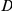. The 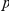-value is the probability of obtaining a smaller 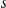 during random permutations. We typically perform 1000 permutations and use a 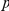-value threshold of 0.002 to determine whether a module and a tree are sufficiently concordant.

### Selecting modules that support common progression

Using Equation (1), we evaluate the statistical concordance between all the gene modules and all the MSTs. Since each MST is constructed based on one gene module, a MST and its corresponding module are concordant by construction. If a module is concordant with the MST derived from another module, these two modules are similar in the sense that they support a common progression pattern.

Based on the statistical concordance between all the modules and all the MSTs, a progression similarity matrix is derived. The 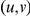 element of the progression similarity matrix is the number of trees that are concordant with both modules 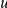 and 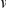. For visualization, we re-order the modules by hierarchical clustering of the columns of the progression similarity matrix [14], so that we can clearly identify similar modules along the diagonal, via visual inspection. We explored several algorithms to automatically identify similar modules from the progression similarity matrix, including hierarchical clustering with gap statistics, spectral partitioning, and forward and backward selection. However, there was not a single algorithm and parameter setting that performed well for all the datasets we analyzed. Since the number of modules in the progression similarity matrix is usually small, we decided to perform module selection manually. An automated algorithm for this step will introduce an additional parameter which is not as intuitive as manual selection. In the progression similarity matrix, if there is a diagonal block whose entries all have relatively high values, i.e. Figure 2(a) and (b), the corresponding modules are similar because they describe a common progression. SPD selects these similar modules, and constructs an overall MST that describes the common progression supported by the selected modules, which is likely to be biologically meaningful.

## Supporting Information

Text S1 Discovering biological progression underlying microarray samples.
(3.56 MB PDF)
