# Supplementary material for: Discovering Biological Progression Underlying Microarray Samples
Source: PLoS Comput Biol. 2011 Apr 14;7(4):e1001123. doi: 10.1371/journal.pcbi.1001123 (PMC3077357; doi:10.1371/journal.pcbi.1001123)
Supplement: Text S1 — Discovering biological progression underlying microarray samples. (3.56 MB PDF) [file pcbi.1001123.s001.pdf]

Supplement Text S1

Discovering Biological Progression underlying Microarray Samples

Peng Qiu[1,2], Andrew J. Gentles[1], Sylvia K. Plevritis[1]

[1]Department of Radiology, Stanford University; [2]Department of Bioinformatics and Computational Biology, University of Texas M.D. Anderson Cancer Center

1. Sample Progression Discovery (SPD) results on cell cycle time series

In cell cycle time series data, the correct order of the samples during the progression of cell cycle was known but not used in SPD. Our goal was to test whether SPD could recover the progression among the samples, and identify genes that reflect the progression. We applied SPD to only samples in one cell cycle, because we did not want SPD to make inference based on the cyclic behavior of cell cycle regulated genes.

Five cell cycle time series are available in (Whitfield et al. 2002). A brief summary of the five time series can be found in the following table.

Datasets	Total number of samples	Number cell cycles covered	Number of samples in the first cycle
1	12	2	7
2	26	3	15
3	48	3	17
4	19	2	11
5	9	1	8

Table S1. Summary of the five cell cycle time series in (Whitfield et al. 2002).

For each of the five datasets, feature selection was performed based on the standard deviation (SD) of each gene. An SD threshold was chosen such that ~3000 genes passed the threshold. SPD clustered genes into co-expressed modules, using an iterative consensus k-means approach. One minimum spanning tree (MST) was constructed based on each module. By evaluating the statistical fit between the modules and the MSTs, SPD constructed a similarity matrix that described the progression similarity between modules, i.e. Figure 2 (a, b) in the main text. Based on the progression similarity matrix, similar modules that shared common progression were identified. SPD then constructed an overall MST to describe the common progression supported by the identified modules. **The common progression pattern and the supporting modules are the outputs of the SPD framework.**

In the following, SPD results of the five time series are shown in an order according to the number of samples in the first cycle.

1.1. SPD applied to cell cycle dataset 3

Dataset 3 contained the largest number of samples in the first cell cycle, 17 samples. We chose an SD threshold to obtain about ~3000 high variance genes. The SD threshold for this dataset was 0.3, and 3196 genes passed the threshold. Clustering parameters were L=200, c_1=0.7, c_2=0.9. SPD recovered the correct time order, and nine associated gene modules. The identified progression order and the mean expression of the nine modules were shown in the main text. We used MSigDB (Subramanian et al. 2005) to annotate the identified modules, as shown in Table S2, where we observed enrichment of cell cycle, E2F targets, etc.

Modules	geneset name	p value
all 9 modules	SERUM_FIBROBLAST_CELLCYCLE	8.77E-38
	BRENTANI_CELL_CYCLE	3.69E-26
	LEE_TCELLS2_UP	3.97E-26
	BRCA_ER_NEG	3.09E-23
	CELL_CYCLE	3.48E-23
	HSA04110_CELL_CYCLE	3.86E-23
	CELL_CYCLE_KEGG	1.68E-19
	LEI_MYB_REGULATED_GENES	2.52E-17
	CROONQUIST_IL6_RAS_DN	8.68E-15
	CELLCYCLEPATHWAY	2.52E-13
Module-3	SERUM_FIBROBLAST_CELLCYCLE	1.22E-34
	HOFFMANN_BIVSBII_BI_TABLE2	2.66E-23
	LEE_TCELLS3_UP	1.60E-21
	TARTE_PLASMA_BLASTIC	4.52E-20
	BRENTANI_CELL_CYCLE	3.79E-19
	LEE_TCELLS2_UP	5.33E-19
	CROONQUIST_IL6_STARVE_UP	5.50E-18
Module-4	AD12_48HRS_DN	3.02E-18
	AD12_ANY_DN	1.00E-17
	AD12_24HRS_DN	7.53E-17
	AD12_32HRS_DN	2.90E-15
	BRCA_ER_NEG	5.62E-13
	BREAST_CANCER_ESTROGEN_SIGNALING	3.81E-12
Module-5	DOX_RESIST_GASTRIC_UP	1.39E-07
	CELL_CYCLE	9.95E-07
	SERUM_FIBROBLAST_CELLCYCLE	4.44E-06
	REN_E2F1_TARGETS	7.21E-05
	LEE_MYC_E2F1_UP	9.38E-05
Module-6	BOQUEST_CD31PLUS_VS_CD31MINUS_UP	6.05E-08
	CMV_HCMV_TIMECOURSE_ALL_DN	6.15E-07
	BROCKE_IL6	9.47E-07
	KRETZSCHMAR_IL6_DIFF	9.47E-07
	UVB_NHEK1_C2	2.61E-06
Module-10	V$E2F1_Q6_01	1.18E-07
	V$E2F1DP1RB_01	4.05E-07
	V$E2F1_Q3	4.05E-07
	SGCGSSAAA_V$E2F1DP2_01	4.05E-07
	V$E2F_Q6	1.14E-06
	V$E2F1_Q6	1.14E-06

Table S2. Gene set annotations of the modules identified by SPD.

1.2. SPD applied to cell cycle dataset 2

The second largest dataset contained 15 samples in the first cell cycle. We used SD threshold 0.4, and obtained 3449 high variance genes. Clustering parameters were L = 200, c_1 = 0.7, c_2 =0.9.

SPD identified five modules that supported a common progression shown in Figure S1. From the middle to the upper right corner, time points 0 ~ 9 were arranged in the correct order. Time point 10 ~ 15 were roughly aligned from the upper left corner to the middle. It appeared that an edge between samples t=9 and t=10 was missing.

Since the samples represented one cell cycle, the starting and ending time points were similar, which explained why samples t=0 and t=15 were connected. Since MST did not allow circles, SPD chose to break the edge between t=9 and t=10 in this example. Therefore, we consider the identified progression tree roughly correct.

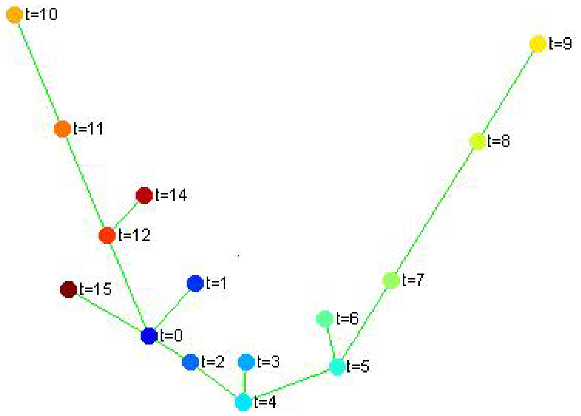

Figure S1. Sample progression order identified by SPD.

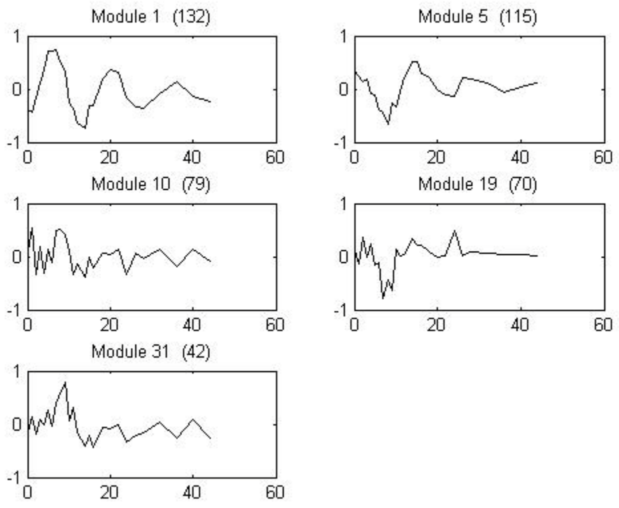

Figure S2. Average expression of the SPD identified modules using data from three cell cycles.

The MSigDB annotations of the identified modules were listed in Table S3. We again observed enrichment of cell cycle and E2F targets. This was quite consistent with the gene set enrichment result in the previous dataset.

all 5 modules	SERUM_FIBROBLAST_CELLCYCLE	2.10E-30
	CELL_CYCLE	2.88E-25
	HSA04110_CELL_CYCLE	4.43E-24
	CELL_CYCLE_KEGG	6.20E-23
	BRENTANI_CELL_CYCLE	1.28E-21
	GAY_YY1_DN	6.83E-20
	IDX_TSA_UP_CLUSTER3	5.17E-18
	TARTE_PLASMA_BLASTIC	9.83E-18
module 1	BRENTANI_CELL_CYCLE	3.90E-16
	SERUM_FIBROBLAST_CELLCYCLE	1.85E-15
	GOLDRATH_CELLCYCLE	3.67E-12
	TARTE_PLASMA_BLASTIC	2.82E-11
	CELL_CYCLE	2.75E-09
	CELL_CYCLE_KEGG	4.47E-09
module 5	V$E2F1_Q6_01	1.07E-19
	CMV_IE86_UP	1.20E-18
	V$E2F_Q3	2.21E-18
	CELL_CYCLE	2.86E-18
	V$E2F1DP1RB_01	3.19E-18
	V$E2F1_Q6	3.59E-18
	V$E2F_Q6	3.59E-18
module 10	V$BRN2_01	1.61E-05
	XU_ATRA_DN	3.22E-05
	HINATA_NFKB_DN	8.63E-05
module 31	LIAN_MYELOID_DIFF_RECEPTORS	3.27E-06
	GCTGAGT,MIR-512-5P	9.21E-06
	CK1PATHWAY	8.14E-05

Table S3. Gene set annotations of the modules identified by SPD.

1.3. SPD applied to cell cycle dataset 4

Dataset 4 contained 11 samples in the first cell cycle. We chose SD threshold 0.45 to obtain 3113 genes with high variance. Clustering parameters were L = 200, c_1 = 0.8, c_2 =0.9.

SPD identified eight gene modules that supported a common progression in Figure S3. The correct time order was perfectly recovered. The mean expressions of the eight modules were computed, in Figure S4.

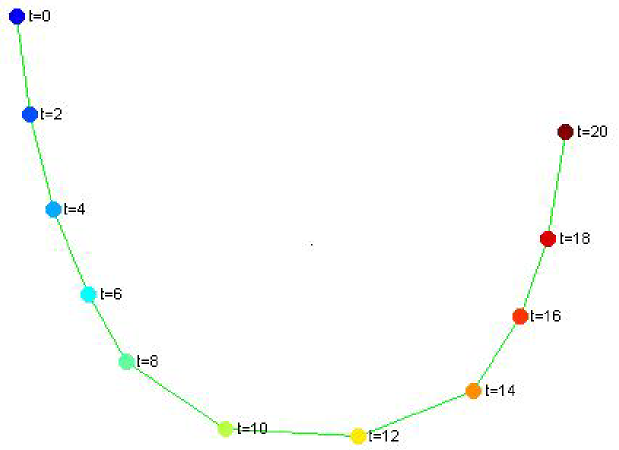

Figure S3. Sample progression order identified by SPD.

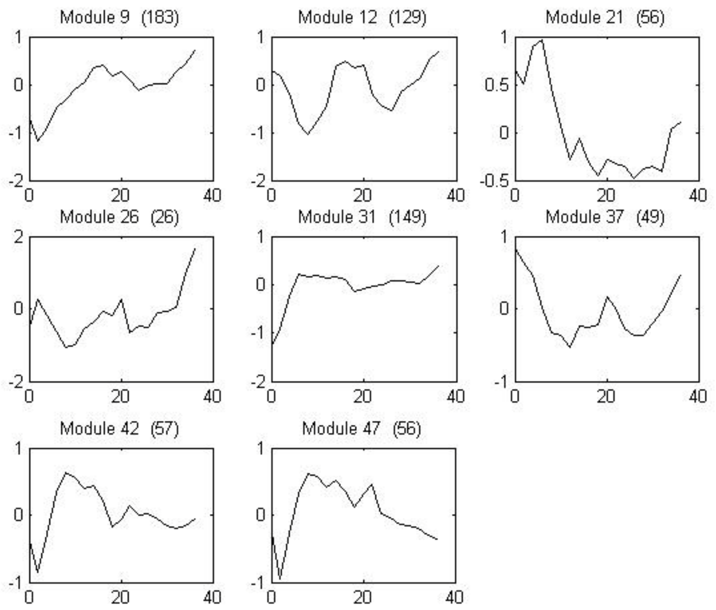

Figure S4. Average expression of the SPD identified modules in two cell cycles.

The eight identified modules were annotated using gene sets in MSigDB, Table S4. Consistent with the results in the previous two cell cycle time series, we observed enrichment of cell cycle genes and E2F targets.

all 8 modules	SERUM_FIBROBLAST_CELLCYCLE	7.81E-42
	LEE_TCELLS2_UP	2.01E-23
	HOFFMANN_BIVSBII_BI_TABLE2	1.19E-21
	TARTE_PLASMA_BLASTIC	7.81E-21
	BRENTANI_CELL_CYCLE	1.38E-19
	CELL_CYCLE	3.81E-18
	LEE_TCELLS3_UP	4.71E-18
	CANCER_UNDIFFERENTIATED_META_UP	7.65E-18
	CELL_CYCLE_KEGG	1.14E-17
	HSA04110_CELL_CYCLE	1.82E-17
module 9	SERUM_FIBROBLAST_CELLCYCLE	6.03E-16
	CMV_IE86_UP	3.21E-11
	VHL_NORMAL_UP	8.56E-09
	CANCER_UNDIFFERENTIATED_META_UP	1.69E-08
	RCC_NL_UP	3.90E-08
	LAMB_CYCLIN_D3_GLOCUS	4.26E-08
module 12	SERUM_FIBROBLAST_CELLCYCLE	4.05E-28
	HOFFMANN_BIVSBII_BI_TABLE2	2.20E-16
	BREAST_DUCTAL_CARCINOMA_GENES	3.87E-16
	LEE_TCELLS3_UP	1.97E-15
	BRENTANI_CELL_CYCLE	2.17E-14
	TARTE_PLASMA_BLASTIC	1.04E-13
	GOLDRATH_CELLCYCLE	1.38E-13
module 21	NELSON_ANDROGEN_UP	4.43E-08
	LEI_MYB_REGULATED_GENES	6.20E-08
	ET743_HELA_UP	4.90E-07
	RUTELLA_HEMATOGFSNDCS_DIFF	6.90E-06
	SHEPARD_NEG_REG_OF_CELL_PROLIFERATION	9.00E-06
module 37	V$E2F1_Q3	5.73E-08
	SGCGSSAAA_V$E2F1DP2_01	1.61E-07
	CMV_IE86_UP	8.34E-07
	KNUDSEN_PMNS_DN	8.40E-07
	V$E2F_Q3	8.62E-07
	V$E2F1_Q4_01	9.30E-07
module 47	HSA04510_FOCAL_ADHESION	7.53E-06
	TGFBETA_ALL_UP	1.89E-05
	GRAEBER_BETA2_INTEGRINS	2.27E-05
	TGFBETA_C4_UP	2.27E-05

Table S4. Gene set annotations of the modules identified by SPD.

1.4. SPD applied to cell cycle datasets 1 and 5

Datasets 1 and 5 were relatively small, containing 7 and 8 samples, respectively. We applied SPD to these two datasets. The identified progression patterns, Figures S5 and S6, were not consistent with the true time order.

The reason is as follows. SPD assumes that the underlying progression pattern can be reflected by the gradual shift in the expression of subsets of genes. If the progression process is not sufficiently probed, meaning that if there are not enough sample points, the time order of the samples can no longer be reflected by the gradual shift in expression. Lack of data points resulted in the inconsistency between the SPD results and the true time order.

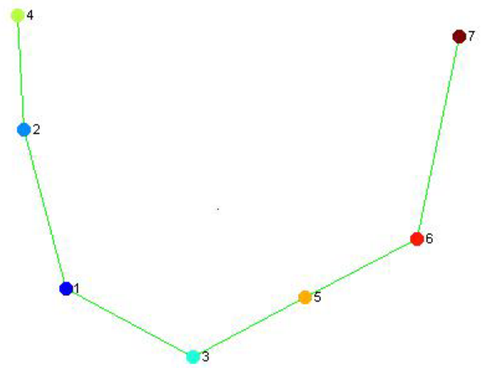

Figure S5. In dataset 1, the progression identified by SPD is not consistent with the time order.

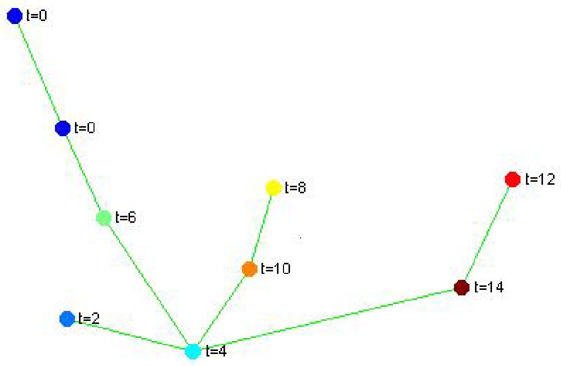

Figure S6. In dataset 5, the progression identified by SPD is not consistent with the time order.

1.5 Robustness of SPD via bootstrap and leave-one-out cross validation

We performed two tests to evaluate the robustness of SPD, bootstrap and leave-one-out cross validation.

In our bootstrap analysis, we used cell cycle dataset 3 as example. We performed 100 iterations of bootstrap. In each of the 100 iterations, 90% of the 3196 genes were randomly selected. SPD was applied to the bootstrapped data. All three steps were performed: clustering, MST construction and statistical comparison. Topological overlap measure (TOM) (Yip and Horvath 2007) distance was used to evaluate the distance between the identified progression and the true time order. The mean TOM distance was 5.36, and the standard deviation was 3.37. The standard deviation of the TOM distance appeared to be comparable to the mean because of the statistical property of this distance metric. The TOM distances of the two worst iterations were 16.2 and 12.7, and the corresponding SPD results were visualized in Figure S7, which were quite consistent with the true time order. Therefore, although the variance of the bootstrapped TOM distance appeared to be large, the identified progression pattern was quite stable during bootstrap.

The statistical significance of the bootstrapped TOM distance should be viewed in conjunction with random permutation analysis. We randomly generated 1000 MSTs, and computed the TOM distance between the random MSTs and the true time order. The mean and standard deviation of the empirical null distribution of TOM was 59.21 ± 8, far away from the TOM distances obtained during bootstrap. Therefore, the TOM distances in all of the 100 bootstrap iterations were significant compared to the random permutation.

To evaluate this statistical significance, we considered permuting the expression data for each gene separately and generate p-value from permuted data. The problem with this approach is as follows: in the progression similarity matrix, we will not observe modules that are similar in terms of progression (the diagonal block in Figure 2 a,b in the main text). Note that the overall MST is constructed based on a set of modules that are similar in terms of progression (the diagonal block). If we permute the data gene by gene, we will not be able to obtain such a diagonal block and MST that are comparable to result from the original data. That is why we decided to randomly generate MSTs to evaluate the significance. We admit that the random trees may not exactly represent the true empirical null distribution. The complexity of the SPD framework makes it difficult to get a null distribution by permuting the data. This is a point that worth further consideration.

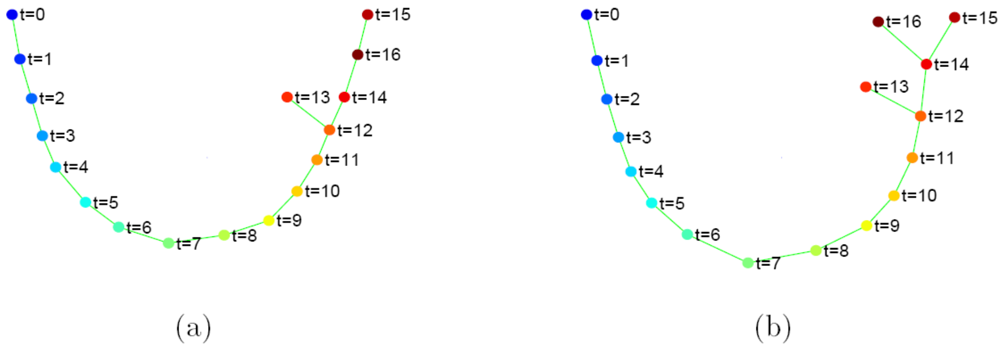

Figure S7. The two bootstrap iterations with worst/largest TOM distance, (a) 16.2 and (b) 12.7

We also performed leave-one-out cross validation (LOOCV). In each of the 17 leave-one-out iterations, we left out one sample, and applied SPD to extract the progression among the remaining samples. When we left out the second sample, for example, we expected SPD to recover the linkage between the first and the third sample. TOM distance was used to evaluate the distance between the SPD identified progression and the true time order. The mean TOM distance was 6.12, and the standard deviation was 4.45.

1.6 Robustness of SPD with respect to the gene clustering component

In practice, when we analyze microarray gene expression data using SPD, we always set L = 200, c2 = 0.9. The choice of the parameter c1 depends on the correlation structure in the data. If there are many gene pairs that share large correlation, we typically set c1 = 0.8. If relatively few gene pairs share high correlation, we set c1 to be smaller, c1 = 0.6, in order to get gene modules of reasonable sizes (at least > 5 genes). In our software implementation of SPD (available at http://icbp.stanford.edu/software/SPD/), we implemented another clustering algorithm, which is agglomerative. This algorithm does not need the parameter L, but it still needs c1 and c2. Again, we set c2=0.9, and choose the value of c1 according to the strength of correlation within the data.

Different choices of clustering parameters will inevitably lead to different gene clustering results. Such differences may propagate into the subsequent steps of SPD and the final progression structure. In this subsection, we evaluate the robustness of SPD with respect to the parameter settings. We also include the agglomerative clustering algorithm in the SPD software in addition to the divisive consensus clustering algorithm described in the Method section of the main text.

We used the cell cycle data behind Figure 1 of the main text for this analysis, varied the algorithm and parameters of the clustering step, and examined the final progression tree. For the divisive consensus clustering algorithm, we set c2 = 0.9, and varied the parameters L and c1. For the agglomerative clustering algorithm, we set c2=0.9, and varied the choice of c1. As expected, different clustering algorithms and parameters produced different gene clusters. However, the final progression tree is robust. In Figures S8-S12, we show the progression similarity matrix and the overall MST for each parameter setting. From these figures, we can observe that the overall MST is reproducible.

Figures S8-S12 only show results of one run for each parameter setting. Since both the divisive and agglomerative clustering algorithms contain randomness, we may get different clustering results in multiple runs with the same parameter setting. The run-to-run variations of the clustering results may also lead to difference in the progression similarity matrices and the overall MSTs. However, we found that the overall MSTs are consistent across multiple runs. This is also shown in the bootstrap analysis in the previous subsection.

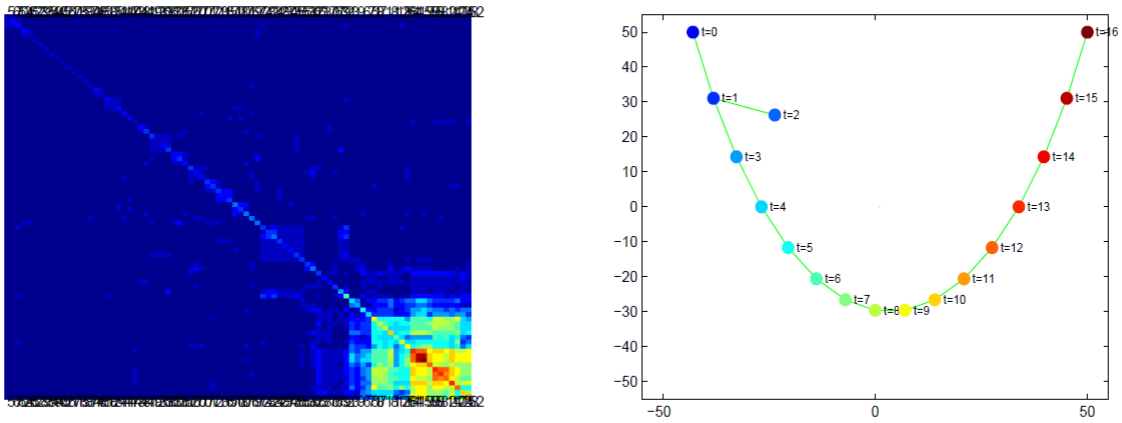

Figure S8. SPD result of the cell cycle dataset. Divisive clustering was performed, with parameters L = 150, c1 = 0.85, c2 = 0.9. The left panel is the progression similarity matrix; the right panel is the overall MST.

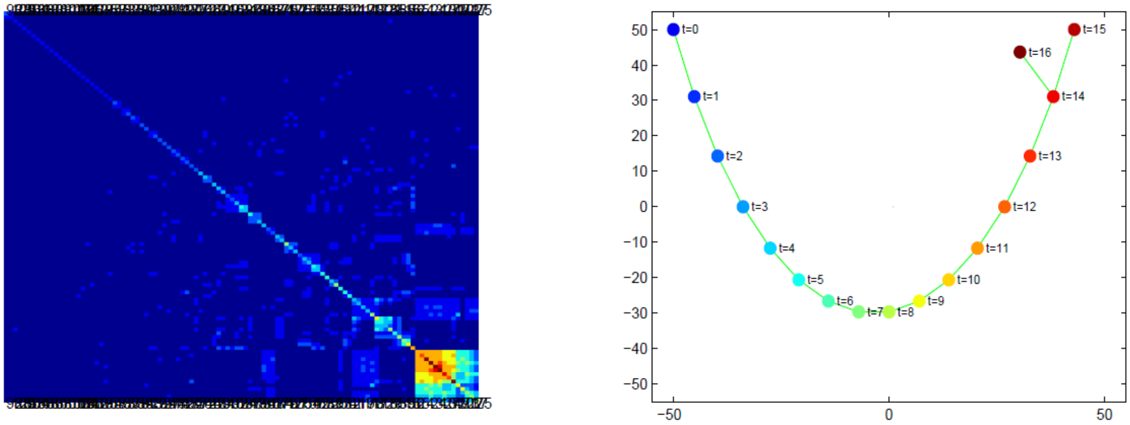

Figure S9. SPD result of the cell cycle dataset. Divisive clustering was performed, with parameters L = 150, c1 = 0.75, c2 = 0.9. The left panel is the progression similarity matrix; the right panel is the overall MST.

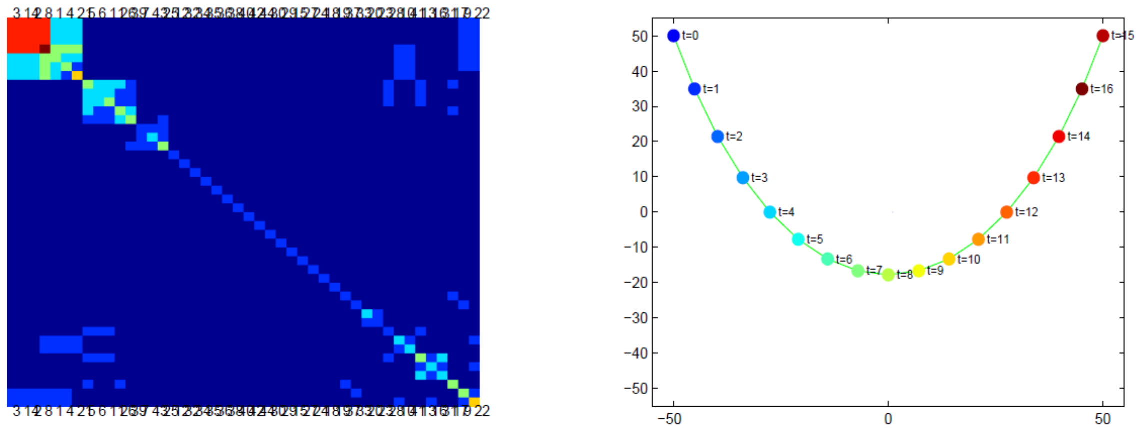

Figure S10. SPD result of the cell cycle dataset. Divisive clustering was performed, with parameters L = 300, c1 = 0.65, c2 = 0.9. The left panel is the progression similarity matrix; the right panel is the overall MST.

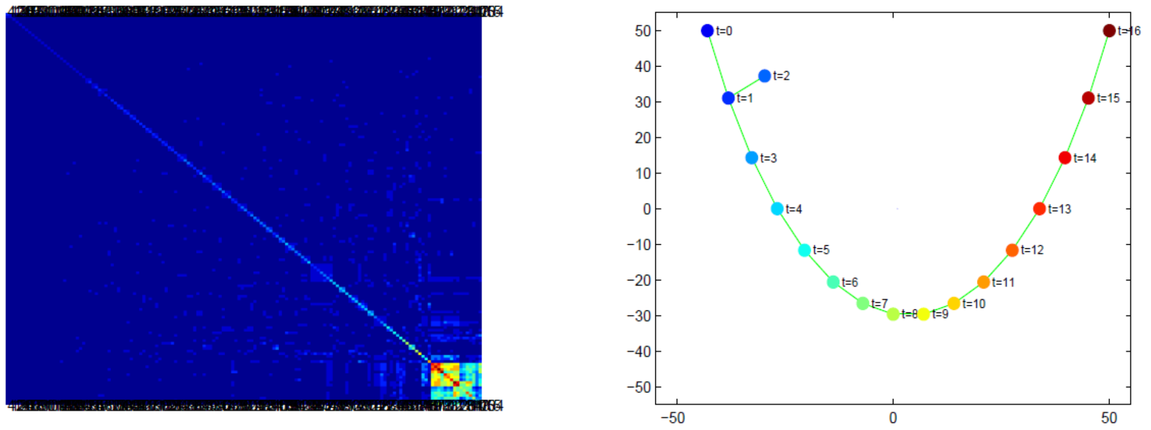

Figure S11. SPD result of the cell cycle dataset. Agglomerative clustering was performed, with parameters c1 = 0.85, c2 = 0.9. The left panel is the progression similarity matrix; the right panel is the overall MST.

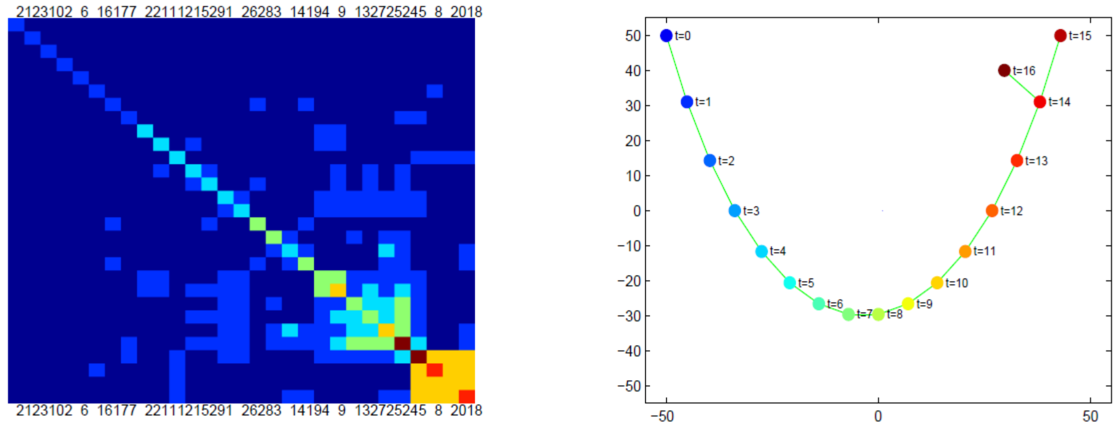

Figure S12. SPD result of the cell cycle dataset. Agglomerative clustering was performed, with parameters c1 = 0.65, c2 = 0.9. The left panel is the progression similarity matrix; the right panel is the overall MST.

1.7 Diameter of the SPD identified progression

SPD can serve as a hypothesis generation tool, when applied to microarray datasets where the progression is unclear or even not exist, i.e. most existing cancer microarray datasets. In such cases, SPD assumes that: cancer development follows a certain progression process; cancer samples collected from individual patients represent different stages of cancer progression; the correct order among the samples may lay out a pathway or trajectory of cancer progression. Under these assumptions, SPD identifies a progression among the samples and gene modules whose gradual shifts reflect the identified progression.

The identified progression is a hypothesis to be tested. Before wet-lab experimental tests, the biological relevance of the SPD result can be assessed by its diameter (defined as the number of edges in the shortest path between the furthest pair of nodes).

For example, we randomly generated 1000 17-node MSTs, and computed their diameters. The mean was 7.7 ± 1.4. In the cell cycle dataset 3, which contained 17 samples, the diameter of the SPD result was 15. The probability of obtaining such a large diameter by chance was very small, which implied that the SPD finding was statistically significant and was likely to be biologically relevant. And indeed, the identified progression was consistent with the progression of cell cycle.

Therefore, when we analyze a dataset where the progression pattern is unknown, if the identified progression has large diameter, it is less likely to happen by chance and thus more likely to be biologically meaningful.

2. SPD results on B-cell differentiation data

module-1	BOQUEST_CD31PLUS_VS_CD31MINUS_UP	0.000180584
	CHIARETTI_T_ALL	0.000164694
	MARTINELLI_IFNS_DIFF	6.31E-05
	CHIARETTI_T_ALL_DIFF	9.69E-05
	VERHAAK_AML_NPM1_MUT_VS_WT_UP	3.09E-05
module-2	Bhattacharya_DownMemB_vs_NaiveB	5.13E-05
	LEE_TCELLS2_UP	1.50E-05
	CAGTGTT,MIR-141,MIR-200A	0.000188404
module-3	SHEPARD_BMYB_MORPHOLINO_UP	0.00016788
	AACTGAC,MIR-223	2.52E-05
module-4	IS_244_GC_B_cell_BL_equal_DLBCL	3.83E-05
module-5	Bhattacharya_UpMemB_vs_GC	8.09E-05
	IS_Hystad_list_Processed	3.51E-05
	KLEIN_PEL_DN	2.67E-05
	Bhattacharya_UpMemB_vs_Plasma	2.73E-07
	WIELAND_HEPATITIS_B_INDUCED	1.66E-09
	Bhattacharya_UpNaiveB_vs_GC	6.10E-05
	BASSO_GERMINAL_CENTER_CD40_UP	4.54E-05
	HSA04662_B_CELL_RECEPTOR_SIGNALING_PATHWAY	9.24E-07
	IS_38_Resting_blood_B_cell_GNF	5.00E-08
	IS_88_Blimp_Bcell_repressed	5.99E-10
	IS_34_Pan_B_U133plus	8.93E-06
	CARIES_PULP_UP	4.17E-07
	BLEO_HUMAN_LYMPH_HIGH_24HRS_UP	2.52E-05
	Bhattacharya_UpNaiveB_vs_Plasma	6.60E-06
	IS_57_CD40_upregulated_Burkitt_lymphoma	2.69E-05
	CARIES_PULP_HIGH_UP	0.000148813
	HSA04612_ANTIGEN_PROCESSING_AND_PRESENTATION	6.71E-05
	SANA_IFNG_ENDOTHELIAL_UP	0.000145208
module-6	IS_Hystad_list_Processed	2.34E-07
	ZHAN_MM_MOLECULAR_CLASSI_DN	1.72E-05
module-7	Fortunel_NPC_vs_RPC	8.09E-05
	ROME_INSULIN_2F_UP	1.52E-06
	HUMAN_MITODB_6_2002	0.000102532
	HSA00190_OXIDATIVE_PHOSPHORYLATION	8.51E-05
	JISON_SICKLECELL_DIFF	9.53E-05
	UVB_NHEK2_UP	3.37E-05
	IFN_BETA_GLIOMA_DN	8.05E-05
	Fortunel_RPC	3.52E-05
	Wong_Mouse_ESC_module	4.68E-05
	IS_10_Proliferation_DLBCL	1.41E-16
	Ben-Porath_Myctargets2	5.55E-05
	Kim_Myc_targets	2.54E-05
	PGC	6.27E-07
	RIBOSOMAL_PROTEINS	8.06E-13
	PENG_RAPAMYCIN_DN	8.01E-06
	MOOTHA_VOXPHOS	3.10E-06
	IS_77_Tcell_cytokine_induced_prolif	4.28E-05
	Chen_Zfx_geneassociations	0.00010474

	Wong_Human_ESC_module	4.17E-08
	CANCER_UNDIFFERENTIATED_META_UP	2.20E-06
	ELECTRON_TRANSPORT_CHAIN	2.06E-05
	MITOCHONDRIA	8.23E-05
	MycTargetDB	4.83E-05
	UVB_NHEK1_UP	3.10E-05
	SC_Ben-Porath_Myctargets2	5.55E-05
	HSA03010_RIBOSOME	4.99E-11
	IS_49_Ribosomal_protein	7.01E-09
	Wong_Core_ESC_module	3.55E-08
	Chen_c-Myc_geneassociations	5.87E-05
module-8	HADDAD_HPCLYMPHO_ENRICHED	1.58E-08
	IS_Hystad_list_Processed	1.43E-04
	IS_56_CD40_downregulated_Burkitt_lymphoma	1.42E-04
	HADDAD_HSC_CD10_UP	5.31E-09
	Schebesta_Pax5_activated	1.62E-05
module-9	HSC_HSCANDPROGENITORS_SHARED	8.08E-06
	HSC_HSCANDPROGENITORS_FETAL	8.08E-06
	HSC_HSCANDPROGENITORS_ADULT	1.08E-05
module-10	IS_Hystad_list_Processed	9.24E-07
	Jensen_CellCycle	7.97E-05
	SC_Ben-Porath_CellCyclinggenes	5.22E-06
	Wong_Mouse_ESC_module	1.19E-06
	Bhattacharya_DownMemB_vs_Plasma	1.93E-05
	GOLDRATH_CELLCYCLE	7.10E-06
	IS_157_Cell_cycle_Liu	7.42E-07
	CROONQUIST_IL6_RAS_DN	8.82E-06
	Ben-Porath_CellCyclinggenes	5.22E-06
	Ben-Porath_Proliferationgenes	1.89E-05
	IS_147_Cell_cycle_Whitfield	3.34E-05
	Bhattacharya_DownNaiveB_vs_GC	3.69E-05
	LEE_TCELLS3_UP	1.04E-06
	SC_Ben-Porath_Proliferationgenes	1.89E-05
	Bhattacharya_DownMemB_vs_GC	1.03E-07

Table S5. Gene set annotations of the SPD identified modules in the B-cell differentiation dataset.

3. SPD identified a landscape of mouse embryonic stem cell differentiation

When applied to mouse embryonic stem cell differentiation data, SPD identified 35 modules that supported a common progression pattern. We annotated modules by comparison to known gene sets in MSigDB, and by examining the relationship between their constituent genes using Ingenuity Pathways Analysis (IPA). In the following, we show the annotations of the 7 modules discussed in the main text, and the progression trees color-coded according to the expression of the 7 modules.

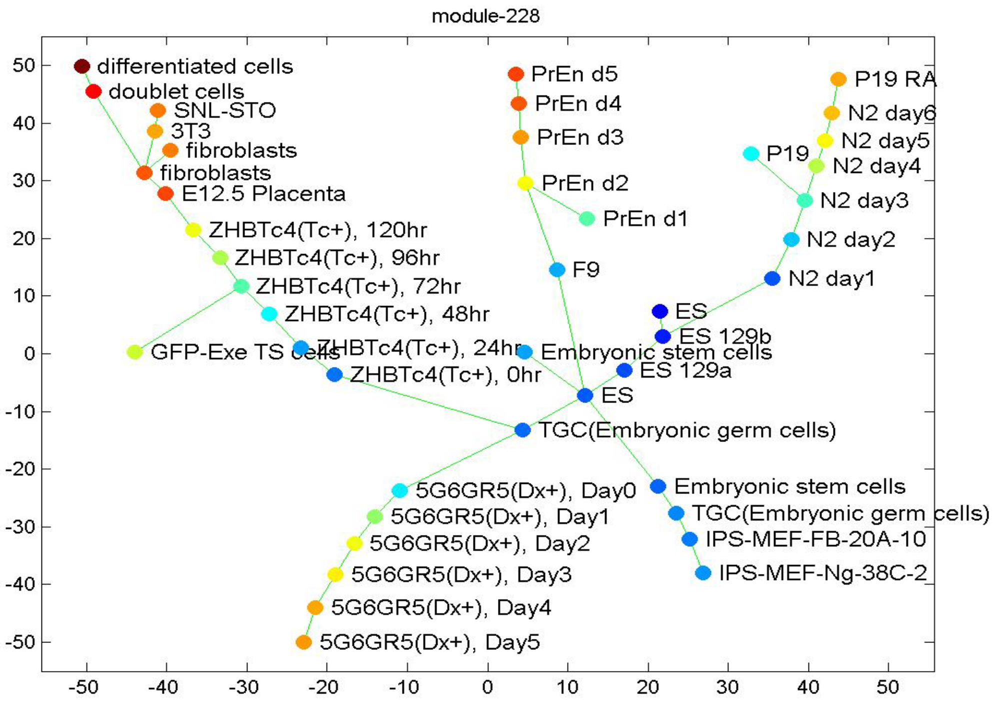

module-228	Chen_Suz12_geneassociations	7.65E-09
	SC_Margueron_Ezh1	1.79E-07
	SC_Margueron_Ezh2	6.79E-06
	Maherali_iPS-ES_methylation_similar	1.04E-05
	Boyer_H3K27_bound	1.47E-05
	SC_Ben-Porath_Suz12targets	1.45E-05
	Ben-Porath_Suz12targets	1.45E-05
	Boyer_Eed_bound	3.55E-05
	Ben-Porath_PRC2targets	6.24E-05
	SC_Ben-Porath_PRC2targets	6.24E-05
	Lee_Suz12_bound	9.31E-05

Figure S13. The SPD identified progression tree color-coded by the mean expression of module-228. Blue means low expression; red means high expression; green/yellow means medium. We observed that module-228 was progressively induced in all differentiating lineages. This module was enriched by targets of Suz12 and Ezh1.

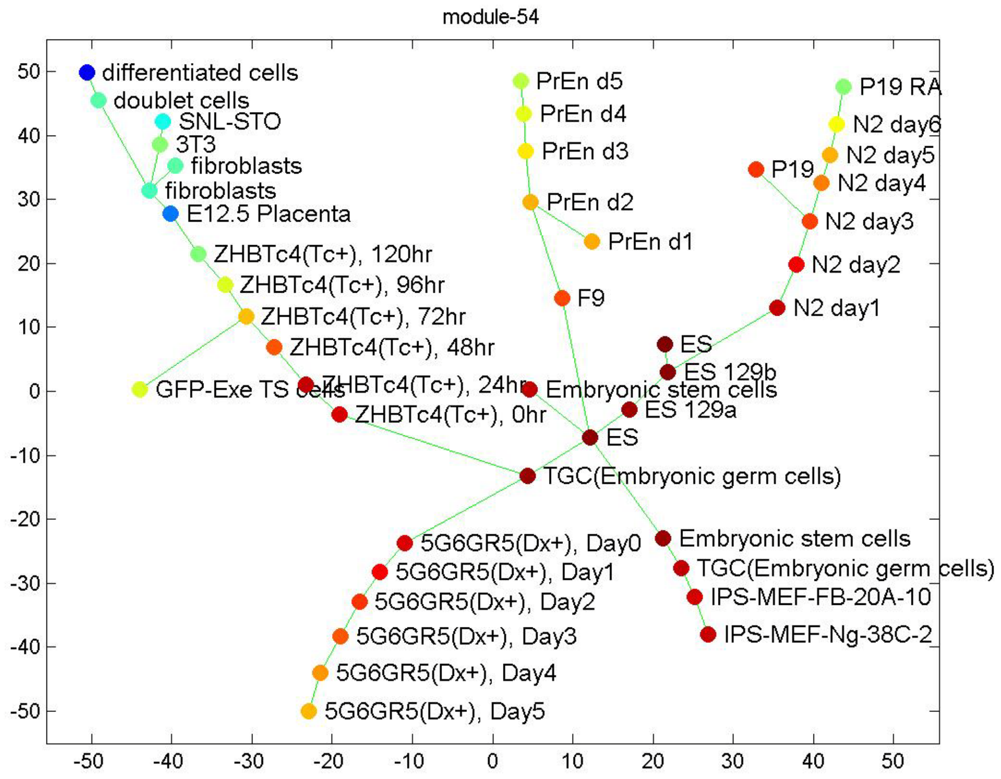

module-54	Kim_Myc_targets	2.58E-10
	Chen_c-Myc_geneassociations	9.42E-10
	Chen_n-Myc_geneassociations	3.35E-09
	Fortunel_ESC	3.62E-06
	Wong_Mouse_ESC_module	2.07E-05
	Chen_E2f1_geneassociations	7.86E-05
	SANSOM_APC_LOSS5_UP	9.04E-05
	STEMCELL_EMBRYONIC_UP	9.96E-05
	IS_167_Myc_overexpression_1.5x_up	0.00016
	IS_168_Myc_overexpression_2x_up	0.000286
	Ivanova_Down_RA_TC	0.000302

Figure S14. Progression color-coded by module-54, which was gradually down-regulated in each differentiating branch. This module was enriched by Myc targets.

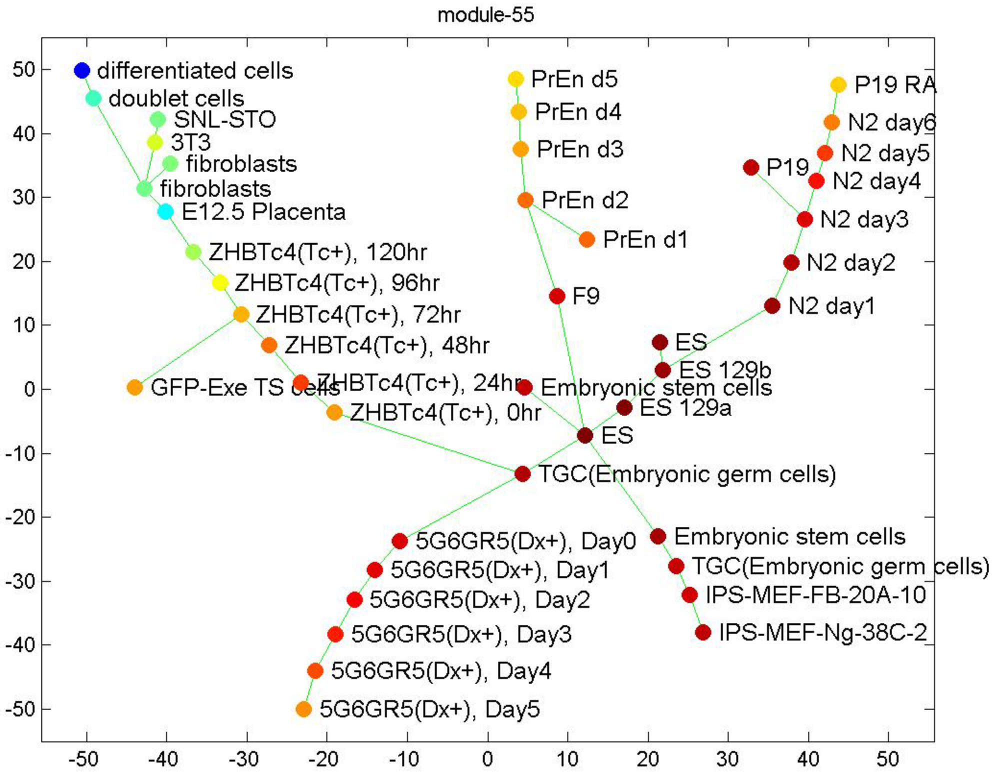

module-55	Kim_Myc_targets	1.68E-07
	Sato_HSC_Enriched_SOURCE	6.85E-07
	Chen_c-Myc_geneassociations	1.29E-06
	Chen_n-Myc_geneassociations	1.11E-05
	HSA00240_PYRIMIDINE_METABOLISM	1.41E-05
	Sato_HSC_MouseAndHuman_enriched_SOURCE	2.79E-05
	Wong_Mouse_ESC_module	3.25E-05
	Fortunel_ESC	4.87E-05
	Wong_Human_ESC_module	0.000108
	FERRANDO_MLL_T_ALL_DN	0.000119
	YU_CMYC_UP	0.000205
	Fortunel_RPC	0.000221
	Chen_E2f1_geneassociations	0.000273
	PYRIMIDINE_METABOLISM	0.000289
	VeneziaHSC_cPsig	0.000342

Figure S15. Progression color-coded by module-55, which was gradually down-regulated in each differentiating branch. This module was enriched by Myc targets and genes involved in Oct4 maintenance of pluripotency.

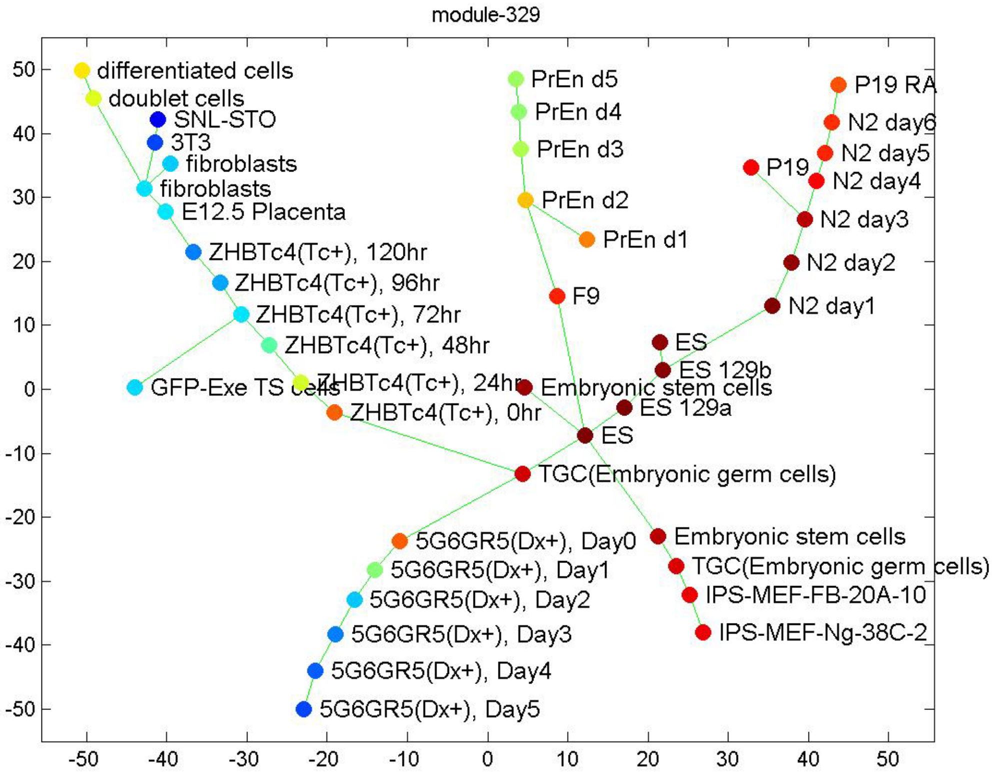

module-329	Bracken_PolycombSuppTable3	2.33E-20
	SC_Bracken_PolycombSuppTable3	2.33E-20
	Lee_H3K27me3_bound	1.11E-09
	Ben-Porath_H3K27bound	1.45E-09
	SC_Ben-Porath_H3K27bound	1.45E-09
	Maherali_ES-MEF_2folddiff	3.22E-09
	IS_96_CNS_PNS_Node1663	1.17E-08
	Ivanova_Down_Pattern2	6.48E-07
	SC_Margueron_Ezh2	8.07E-07
	Chen_Suz12_geneassociations	6.23E-06
	Mathur_Nanog4KO_Down	7.30E-06
	Boyer_H3K27_bound	8.49E-05
	Mathur_Oct4KO_Down	0.000123
	Lee_Eed_bound	0.000168
	TAKEDA_NUP8_HOXA9_10D_UP	0.00027
	STRIATED_MUSCLE_CONTRACTION	0.000459
	Ben-Porath_Eedtargets	0.000477
	SC_Ben-Porath_Eedtargets	0.000477

Figure S16. Progression color-coded by module-329, which was progressively down-regulated in all lineages except the neural lineage, which suggested particular subsets of tissue-specific genes. This module was enriched by targets of the Ezh2/Polycomb complex.

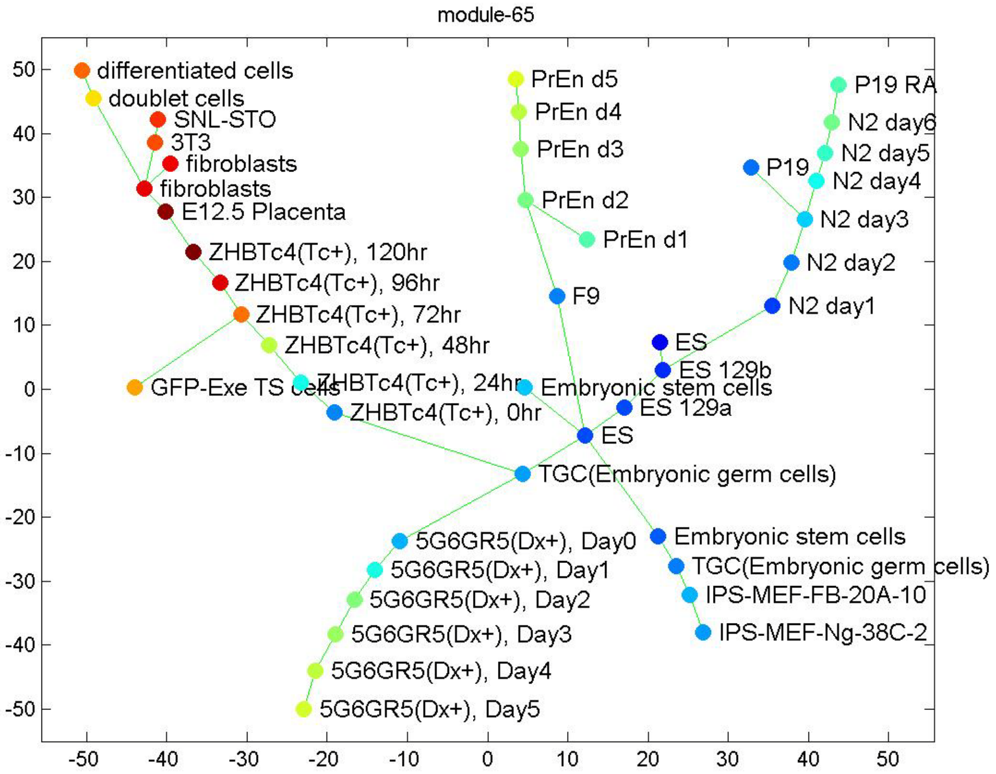

module-65	Ivanova_Nanog_shRNA	2.80E-07
	Krivtsov_HSC_Up_vs_normal	4.35E-07
	Mikkelsen_Down_iPS-ES	1.17E-06
	Ivanova_Oct4_shRNA	2.47E-06
	Wu_Myc_ReversiblyInduced	4.60E-06
	Wong_Mouse_AdultSC_module	6.18E-06
	Ivanova_Up_Pattern2	1.01E-05
	SC_Margueron_Ezh2	1.05E-05
	Ivanova_AffectedOne_shRNA_TC	1.29E-05
	Mathur_Oct4KO_Up	1.65E-05
	Wu_Myc_PermInduced	6.20E-05
	Boyer_H3K27_bound	7.47E-05
	IS_31_Monocyte_4x_U133plus	0.000109
	Krivtsov_LeukemicGMP_Up_vs_normal	0.000165
	CMV_24HRS_DN	0.000291

Figure S17. Progression color-coded by module-65, which was strongly induced in trophoblast differentiation, and modestly in the other branches. This module contained numerous genes that are induced by shRNA knockdown Sox2, as well as apoptosis-related genes.

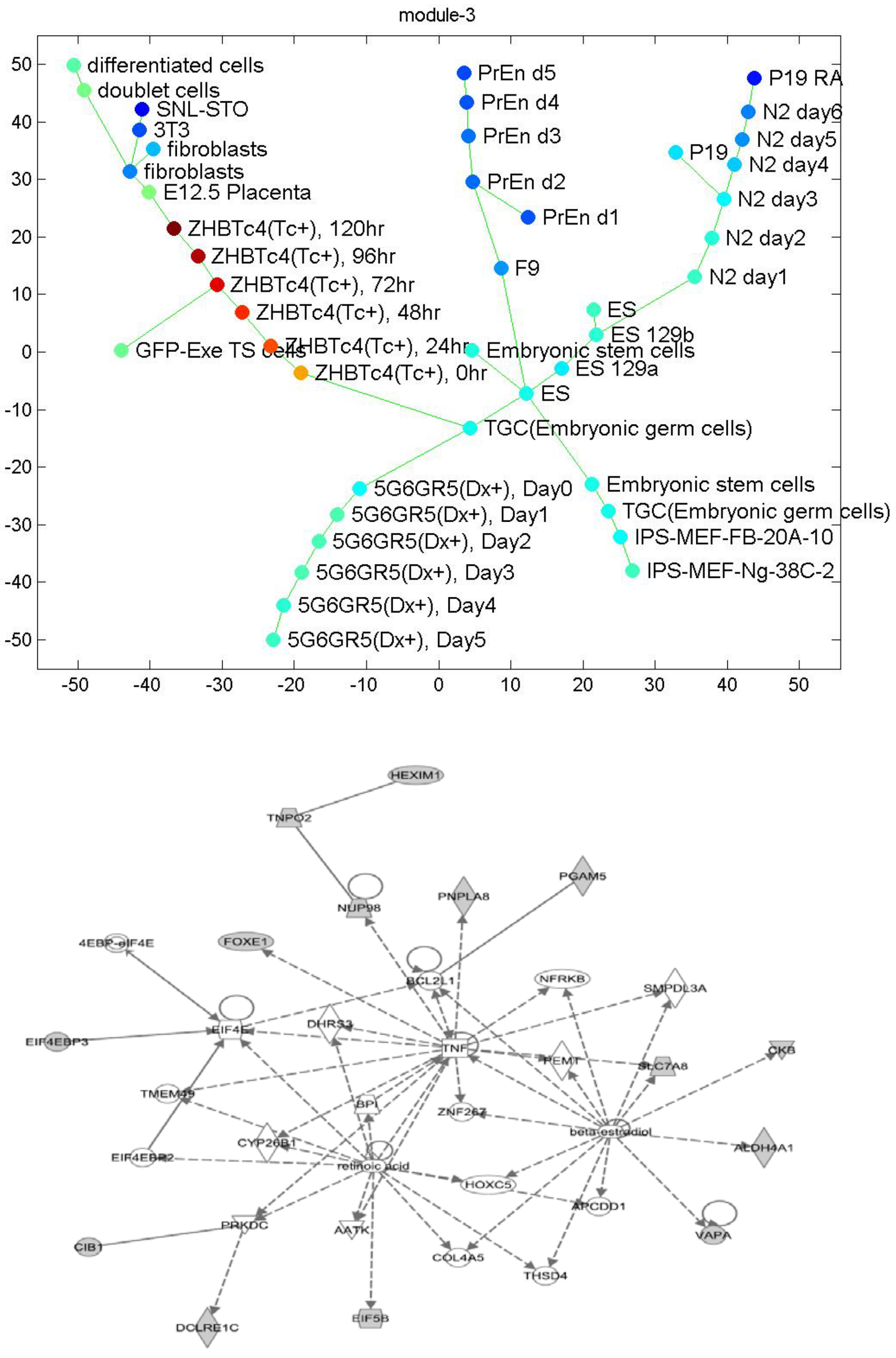

Figure S18. Progression color-coded by module-3, which was highly specifically regulated along the trophoblast differentiation branch. IPA analysis indicated that this module was highly enriched with targets of tumor necrosis factor TNF, (gray nodes are genes in module-3 that are close to TNF).

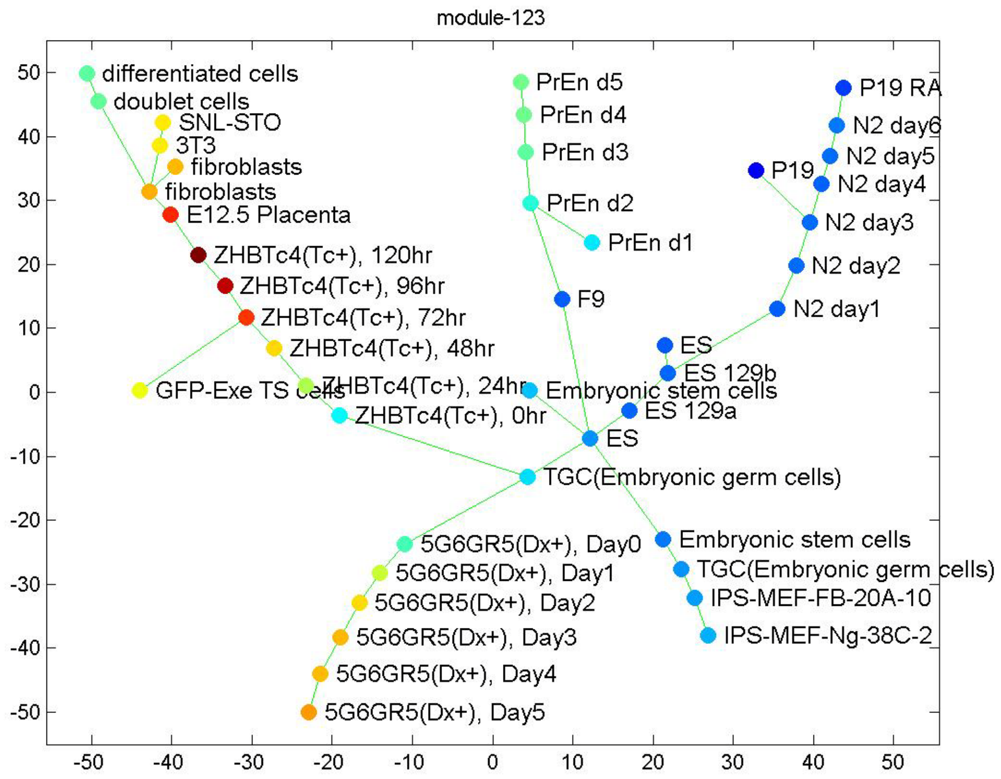

module-123	CELL_MOTILITY	3.51E-05
	Monocyte_T0_UP	0.000154
	ACTINYPATHWAY	0.000256

Figure S19. Progression color-coded by module-123, which was highly specifically regulated along the trophoblast differentiation branch. This module was enriched by cell motility genes, which was consistent with the invasive character of trophoblasts.

4. SPD applied to a prostate cancer dataset

We applied SPD to a prostate cancer microarray dataset (GSE6919). This dataset contains normal prostate tissue free of any pathological alteration from organ donor, normal prostate tissue adjacent to tumor (NAP), tumor samples, and metastatic prostate tumor samples (Mets). An arbitrary standard deviation threshold of 0.6 was chosen to select genes that have high standard deviation. 5670 genes passed the threshold. We clustered the genes using an agglomerative algorithm implemented in our software (http://icbp.stanford.edu/software/SPD/). The clustering parameters were: c1 = 0.7, c2 = 0.9. In this dataset, the average correlation between genes was fairly small, which led to small modules. We excluded modules that contained less than 5 genes. After the clustering step, we obtained 46 modules that were coherent and had more than 5 genes. The total number of genes in these modules was 1007. SPD selected 12 modules (487 genes) that shared high progression similarity and derived a tree structure shown in Figure S20. Normal and metastatic samples are enriched at the left and right ends of the tree. Although NAP and tumor samples are mixed in the middle, NAP samples are more enriched near normal samples, while tumor samples are more enriched near the metastatic samples. The mix of NAP and tumor samples reflects possible field effect suggested by Chandran et al, 2005 in BMC Cancer: normal tissue adjacent to primary tumor is more similar to tumor than it is to normal tissues. This tree structure reflects the general trend we expected. In addition to this general trend, we also observe details that we did not expect to see: i.e. the normal samples mixed with NAP and tumor samples, the two branches of metastatic samples.

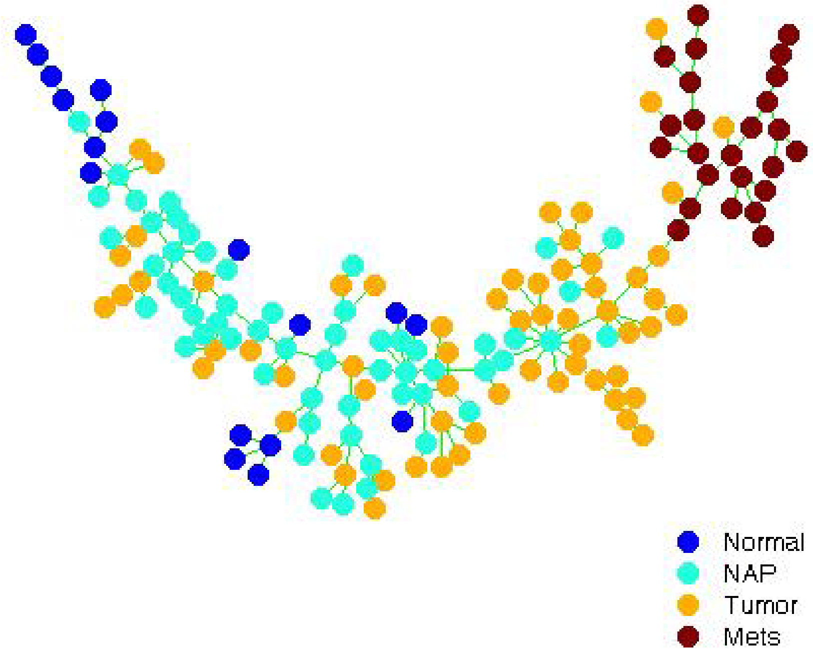

Figure S20. SPD applied to a prostate cancer microarray dataset, GSE6919.

We color coded this tree structure using the average gene expression level of each of the 12 modules, and observed several expression patterns across the tree. In Figure S21, we show 4 modules that exhibit a similar pattern, high in normal and NAP samples, mid/high in tumor, and low in Mets. Gene set enrichment annotations of these 4 modules are listed in Table S6.

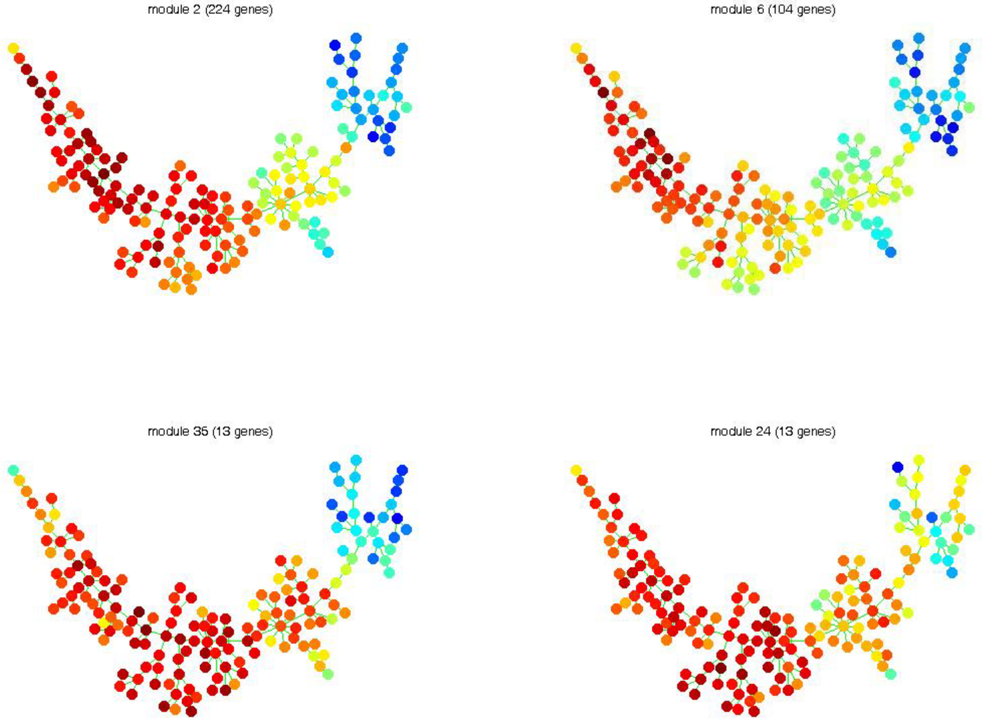

Figure S21. SPD derived tree structure, color-coded by 4 of the 12 selected modules.

Geneset name	Description	pvalue
WEST_ADRENOCORTICAL_TUMOR_DN	Down-regulated genes in pediatric adrenocortical tumors (ACT) compared to the normal tissue.	0
CHARAFE_BREAST_CANCER_LUMINAL_VS_MESENCHYMAL_DN	Genes down-regulated in luminal-like breast cancer cell lines compared to the mesenchymal-like ones.	0
LINDGREN_BLADDER_CANCER_CLUSTER_2B	Genes specifically up-regulated in Cluster IIb of urothelial cell carcinom (UCC) tumors.	0
VECCHI_GASTRIC_CANCER_EARLY_DN	Down-regulated genes distinguishing between early gastric cancer (EGC) and normal tissue samples.	0
SABATES_COLORECTAL_ADENOMA_DN	Genes down-regulated in colorectal adenoma compared to normal mucosa samples.	0
DELYS_THYROID_CANCER_DN	Genes down-regulated in papillary thyroid carcinoma (PTC) compared to normal tissue.	0
TURASHVILI_BREAST_DUCTAL_CARCINOMA_VS_DUCTAL_NORMAL_DN	Genes down-regulated in ductal carcinoma vs normal ductal breast cells.	0
BERTUCCI_MEDULLARY_VS_DUCTAL_BREAST_CANCER_DN	Genes down-regulated in medullary breast cancer (MBC) relative to ductal breast cancer (DBD).	0
LIU_PROSTATE_CANCER_DN	Genes down-regulated in prostate cancer samples.	0
TURASHVILI_BREAST_DUCTAL_CARCINOMA_VS_LOBULAR_NORMAL_DN	Genes down-regulated in ductal carcinoma vs normal lobular breast cells.	0
CHANDRAN_METASTASIS_DN	Genes down-regulated in metastatic tumors from the whole panel of patients with prostate cancer.	0

Table S6. Gene set enrichment of all genes in the 4 modules shown in Figure S21.

The 5 modules in Figure S22 show a different expression pattern: low in normal and Mets, relatively high in NAP and tumor samples. The expression patterns in the middle part of the tree indicate that the NAP and tumor samples can be separated into three groups. This separation can also be observed in Figures S21 and S23.

Another interesting observation is that, modules 17, 19 and 41 are show clear difference between the two branches in the upper right corner, which correspond to the metastatic samples. In this dataset, the metastatic samples were taken from liver, lymph nodes, adrenal gland, or kidney. The two branches do not correlate with the origin of the metastatic samples.

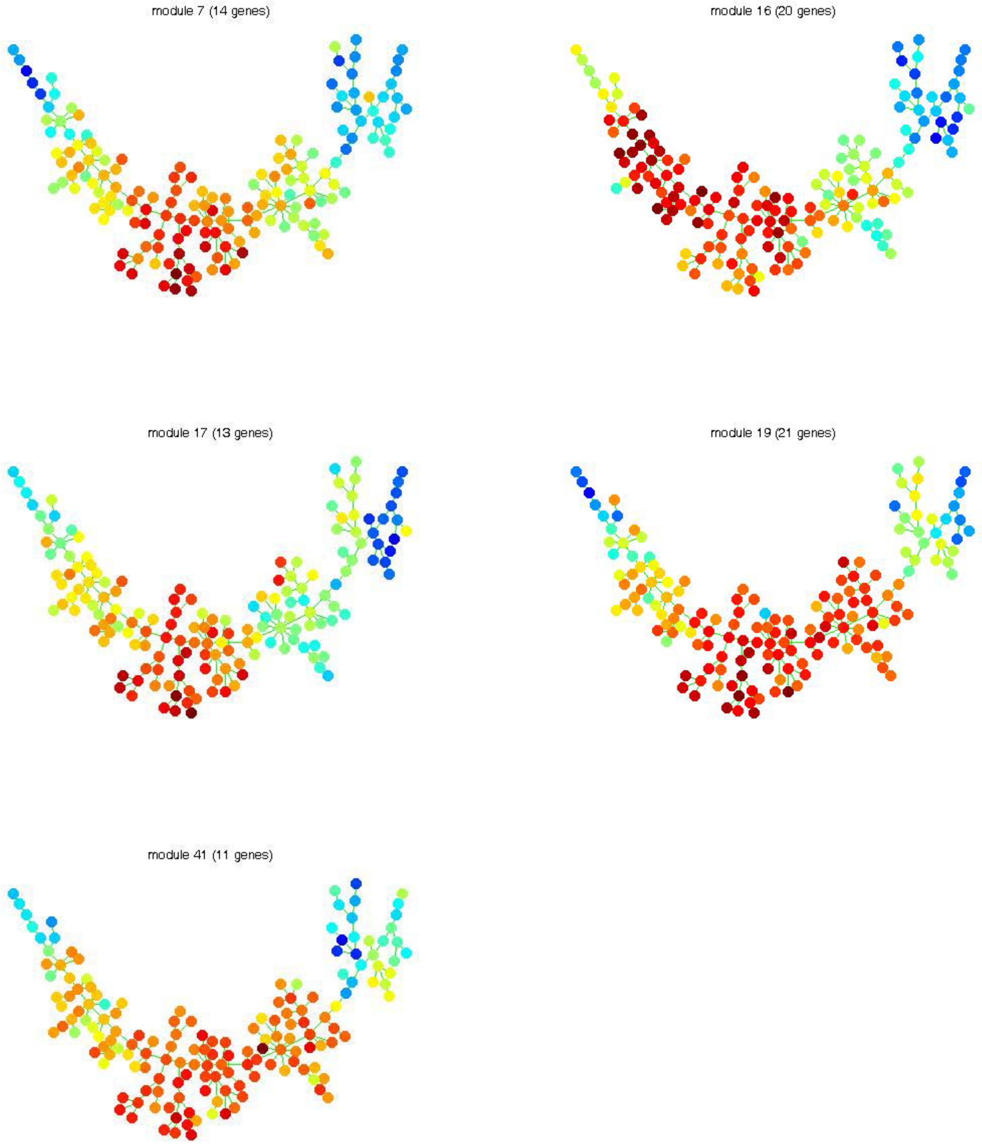

Figure S22. SPD derived tree structure, color-coded by 3 of the 12 selected modules.

Geneset name	Description	pvalue
CHANDRAN_METASTASIS_DN	Genes down-regulated in metastatic tumors from the whole panel of patients with prostate cancer.	2.80E-11
JAEGER_METASTASIS_DN	Genes down-regulated in metastases from malignant melanoma compared to the primary tumors.	6.93E-11
ONDER_CDH1_TARGETS_2_DN	Genes down-regulated in HMLE cells (immortalized nontransformed mammary epithelium) after E-cadhedrin (CDH1) knockdown by RNAi.	4.95E-09
COLDREN_GEFITINIB_RESISTANCE_DN	Genes down-regulated in NSCLC (non-small cell lung carcinoma) cell lines resistant to gefitinib compared to the sensitive ones.	6.56E-08
CHARAFE_BREAST_CANCER_BASAL_VS_MESENCHYMAL_UP	Genes up-regulated in basal-like breast cancer cell lines as compared to the mesenchymal-like ones.	1.75E-07
CHARAFE_BREAST_CANCER_LUMINAL_VS_MESENCHYMAL_UP	Genes up-regulated in luminal-like breast cancer cell lines compared to the mesenchymal-like ones.	3.10E-07
DELYS_THYROID_CANCER_UP	Genes up-regulated in papillary thyroid carcinoma (PTC) compared to normal tissue.	8.09E-07
CHARAFE_BREAST_CANCER_LUMINAL_VS_BASAL_DN	Genes down-regulated in luminal-like breast cancer cell lines compared to the basal-like ones.	2.62E-06
WU_CELL_MIGRATION	Genes associated with migration rate of 40 human bladder cancer cells.	2.84E-06
SABATES_COLORECTAL_ADENOMA_UP	Genes up-regulated in colorectal adenoma compared to normal mucosa samples.	6.64E-06
MCBRYAN_PUBERTAL_BREAST_3_4WK_UP	Genes up-regulated during pubertal mammary gland development between weeks 3 and 4.	2.80E-06

Table S7. Gene set enrichment of all genes in the 4 modules shown in Figure S22.

Although it is not surprising that genes previously identified as being down-regulated in metastases vs primary tumors show the same pattern here, it was unexpected that they were also less highly expressed in normal tissues than in primary tumors. Since these modules overlapped with changes in gene expression involved in metastasis in several epithelial cancers (not just other prostate studies), they may reflect general processes underlying the epithelial-mesenchymal transition and cell migration. Of note, one of the genes in this module was CDH3, a member of the cadherin family that interacts with CDH1. Targeted down-regulation of cadherins by RNA interference has been demonstrated to induce cell migration. However, up-regulation from normal to primary tumors followed by down-regulation in metastases has not been commented upon previously to our knowledge.

We also applied IPA to the list of genes that comprised these modules. The most significant interaction network (p=1e-37) centered around genes involved in androgen and estrogen signaling, and influenced by beta-estradiol. Although estradiol is the predominant sex hormone in females, it is also produced in males as a metabolic product of testosterone. Androgen signaling generally has a pro-survival effect in prostate cancers. Thus one possible interpretation of the SPD result is that it reflects the fact that in primary tumors, androgen signaling up-regulation confers a selective advantage in the natural history of the tumor; but that some metastases develop androgen-independence. A priori, from gene expression profiles, it is unknown which metastases are androgen-independent; hence SPD may be identifying both androgen-independent samples, together with the genes whose changes in expression drive the phenomenon.

The rest 3 of the 12 selected modules are shown in Figure S23. These three modules show high expression in the metastatic samples, while changes among other sample classes are relatively subtle. In Figure S23, we again observe the difference between the two branches that correspond to metastatic samples, and variation of expression in the middle part of the tree which corresponds to NAP and tumor samples.

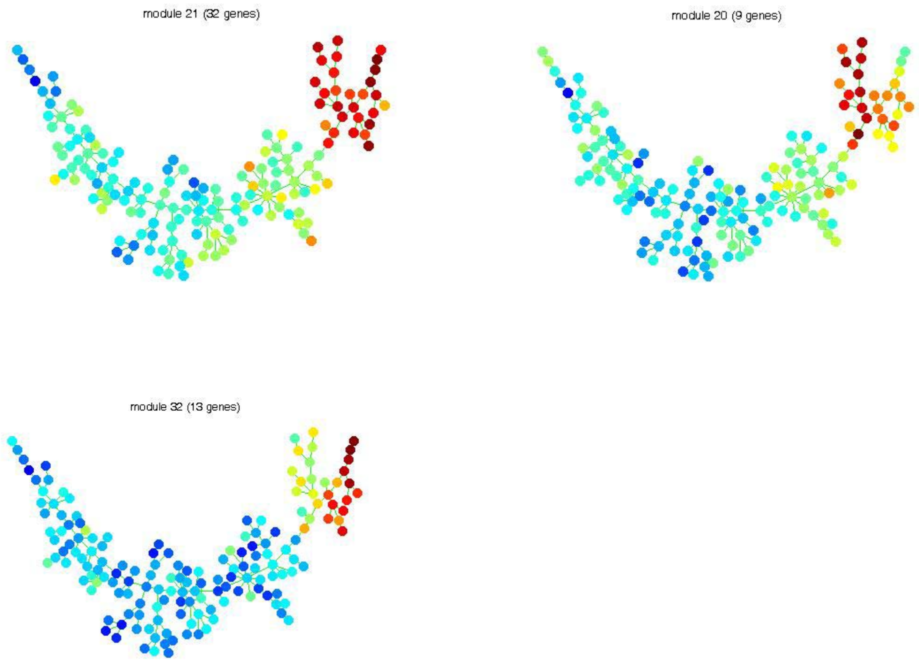

Figure S23. SPD derived tree structure, color-coded by 3 of the 12 selected modules.

Geneset name	Description	pvalue
SHEDDEN_LUNG_CANCER_POOR_SURVIVAL_A6	Cluster 6 of method A: up-regulation of these genes in patients with non small cell lung cancer (NSCLC) predicts poor survival outcome.	7.66E-11
ROSTY_CERVICAL_CANCER_PROLIFERATION_CLUSTER	The 'Cervical Cancer Proliferation Cluster' (CCPC): genes whose expression in cervical carcinoma positively correlates with that of the HPV E6 and E7 oncogenes; they are also differentially expressed according to disease outcome.	3.42E-10
SOTIRIOU_BREAST_CANCER_GRADE_1_VS_3_UP	Up-regulated genes whose expression correlated with histologic grade of invasive breast cancer tumors: comparison of grade 1 vs grade 3.	6.96E-10
CHANDRAN_METASTASIS_UP	Genes up-regulated in metastatic tumors from the whole panel of patients with prostate cancer.	9.19E-10
VECCHI_GASTRIC_CANCER_EARLY_UP	Up-regulated genes distinguishing between early gastric cancer (EGC) and normal tissue samples.	1.20E-08
CROONQUIST_IL6_DEPRIVATION_DN	Genes down-regulated in the ANBL-6 cell line (multiple myeloma, MM) after withdrawal of IL6.	1.26E-08
KOBAYASHI_EGFR_SIGNALING_24HR_DN	Genes down-regulated in non-small cell lung cancer resistant to gefitinib after treatment with EGFR inhibitor CL-387785 for 24h.	3.50E-08
CHANG_CYCLING_GENES	Fibroblast serum response genes showing periodic expression during the cell cycle; excluded from the core serum response signature.	5.73E-08
PUJANA_BRCA2_PCC_NETWORK	Genes constituting the BRCA2-PCC network of transcripts whose expression positively correlated (Pearson correlation coefficient, PCC >= 0.4) with that of BRCA2 [Gene ID=675] across a compendium of normal tissues.	1.66E-07
RUIZ_TNC_TARGETS_DN	Genes down-regulated in T98G cells (glioblastoma) by TNC.	4.07E-07

Table S8. Gene set enrichment of all genes in the 4 modules shown in Figure S23.

5. SPD applied to microarray dataset of FL-DLBCL transformation

Follicular lymphoma (FL) is a relatively indolent B-cell malignancy that frequently undergoes histological transformation to aggressive diffuse large B-cell lymphoma (DLBCL), with drastically worse patient prognosis. We used SPD to analyze a dataset consisting of 24 paired samples of FL and DLBCL, obtained from 12 patients before and after transformation Glas, et al 2005. Without using the class information of the aggressiveness of the samples, SPD identified seven modules (426 genes in total) that fit well with a common progression pattern. As shown in Figure S24, the FL and DLBCL samples were perfectly separated. We used the canonical pathways in the Molecular Signatures Database (MSigDB) to annotate the 426 genes. The annotation results were listed in Table S9. We noticed enrichment of proliferation genes and embryonic stem cell genes. If we order samples from left to right, we observe a gradual increase of the expression of both the proliferation genes and the embryonic stem cell genes. This observation is consistent with our understanding that DLBCL is more highly proliferative than FL. This example further demonstrates that SPD is able to determine which features are relevant to the progression. Moreover, the connection with stem-cell related transcriptional programs resonates with recent findings in FL and other hematological malignancies, implicating a role for them in cancer progression and aggressiveness, Ben-Porath et al 2008, Wong et al 2008, Gentles et al 2009.

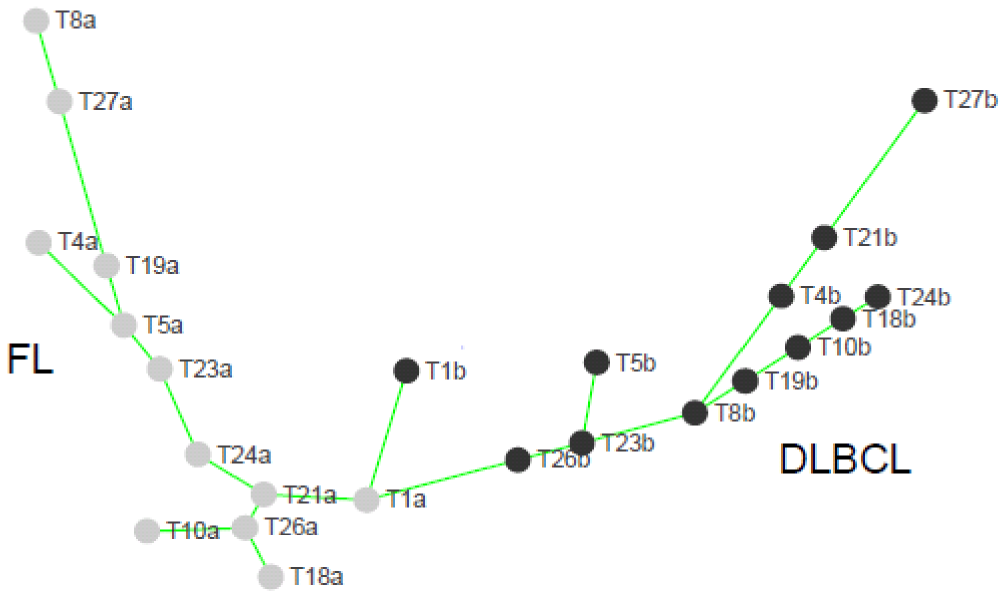

Figure S24. SPD applied to FL and DLBCL samples. FL samples are colored gray. DLBCL samples are colored black. T1a is FL sample from patient 1; T1b corresponds to DLBCL samples from patient 1.

all 7 modules	STEMCELL_NEURAL_UP	7.22E-41
	TARTE_PLASMA_BLASTIC	1.70E-37
	SERUM_FIBROBLAST_CELLCYCLE	1.64E-36
	CANCER_UNDIFFERENTIATED_META_UP	9.81E-34
	LEE_TCELLS2_UP	4.44E-33
	STEMCELL_EMBRYONIC_UP	1.86E-30
	BRCA_ER_NEG	3.02E-28
	SERUM_FIBROBLAST_CORE_UP	6.11E-25
	LI_FETAL_VS_WT_KIDNEY_DN	2.13E-23
	CHANG_SERUM_RESPONSE_UP	3.09E-23
module 1	SERUM_FIBROBLAST_CELLCYCLE	3.12E-29
	LEE_TCELLS3_UP	9.27E-23
	ZHAN_MM_CD138_PR_VS_REST	9.96E-21
	CANCER_UNDIFFERENTIATED_META_UP	1.37E-18
	LI_FETAL_VS_WT_KIDNEY_DN	5.67E-18
	LEE_TCELLS2_UP	9.55E-18
	IDX_TSA_UP_CLUSTER3	5.39E-17
	BRCA_ER_NEG	3.88E-16
	DOX_RESIST_GASTRIC_UP	1.94E-15
	P21_P53_ANY_DN	6.32E-15
module 2	PENG_GLUTAMINE_DN	2.01E-08
	FLECHNER_KIDNEY_TRANSPLANT_WELL_UP	4.19E-06
	CHANG_SERUM_RESPONSE_UP	4.33E-06
	TMTCGCGANR_UNKNOWN	8.01E-06
module 3	CANCER_UNDIFFERENTIATED_META_UP	2.33E-09
	LEE_TCELLS2_UP	4.23E-09
	STEMCELL_NEURAL_UP	2.23E-07
	HDACI_COLON_BUT_DN	2.38E-07
	BRENTANI_CELL_CYCLE	4.22E-07
	TARTE_PLASMA_BLASTIC	1.05E-06
	OLDAGE_DN	1.43E-06
	ET743_SARCOMA_72HRS_DN	2.39E-06
	IRITANI_ADPROX_LYMPH	2.89E-06
	ET743_SARCOMA_DN	7.13E-06
module 4	MANALO_HYPOXIA_DN	8.28E-17
	CANCER_NEOPLASTIC_META_UP	1.32E-12
	DNA_REPLICATION_REACTOME	1.18E-11
	CMV_IE86_UP	1.59E-11
	CHANG_SERUM_RESPONSE_UP	3.74E-11
	SGCGSSAAA_V$E2F1DP2_01	1.28E-10
	SERUM_FIBROBLAST_CORE_UP	6.38E-10
	SERUM_FIBROBLAST_CELLCYCLE	6.89E-10
	LEE_TCELLS2_UP	1.50E-09

	STEMCELL_EMBRYONIC_UP	1.90E-09
module 5	ONE_CARBON_POOL_BY_FOLATE	4.93E-08
	STEMCELL_NEURAL_UP	5.11E-08
	HSA00670_ONE_CARBON_POOL_BY_FOLATE	6.56E-08
	TARTE_PLASMA_BLASTIC	1.01E-06
	BREASTCA_TWO_CLASSES	1.40E-06
	PENG_RAPAMYCIN_DN	1.67E-06
	STEMCELL_EMBRYONIC_UP	3.88E-06
module 6	STEMCELL_NEURAL_UP	4.94E-06

Table S9. Gene set annotations of the SPD identified modules in FL-DLBCL transformation dataset.
